# Checklist of the Sarcophagidae (Diptera) of Croatia, with new records from Croatia and other Mediterranean countries

**DOI:** 10.3897/zookeys.831.30795

**Published:** 2019-03-18

**Authors:** Stjepan Krčmar, Daniel Whitmore, Thomas Pape, Eliana Buenaventura

**Affiliations:** 1 Department of Biology, Josip Juraj Strossmayer University of Osijek, Cara Hadrijana 8/A, HR-31000 Osijek, Croatia Josip Juraj Strossmayer University of Osijek Osijek Croatia; 2 Department of Entomology, Staatliches Museum für Naturkunde, Rosenstein 1, 70191 Stuttgart, Germany Department of Entomology, Staatliches Museum für Naturkunde Stuttgart Germany; 3 Natural History Museum of Denmark, University of Copenhagen, Universitetsparken 15, DK-2100 Copenhagen, Denmark University of Copenhagen Copenhagen Denmark; 4 Museum für Naturkunde, Leibniz Institute for Evolution and Biodiversity Science, Invalidenstraße 43, 10115 Berlin, Germany Museum für Naturkunde, Leibniz Institute for Evolution and Biodiversity Science Berlin Germany

**Keywords:** Balkans, Bulgaria, flesh flies, Greece, Italy, new synonyms, Sardinia, Sicily, Southeast Europe

## Abstract

An updated checklist of Croatian flesh flies is presented based on the literature, on material collected from 2004 to 2017, and on specimens in museum collections. The checklist comprises 22 genera and 148 species (two left unnamed), 105 of which are represented by new Croatian records. Twenty-five species are recorded from Croatia with certainty for the first time: *Amobiapelopei* (Rondani, 1859), *Apodacraseriemaculata* Macquart, 1854, *Craticulinatabaniformis* (Fabricius, 1805), *Macronychiastriginervis* (Zetterstedt, 1838), *Metopiacampestris* (Fallén, 1810), *Miltogrammabrevipila* Villeneuve, 1911, *Miltogrammaiberica* Villeneuve, 1912, *Miltogrammaoestracea* (Fallén, 1820), *Miltogrammapunctata* Meigen, 1824, *Oebalia cylindrica* (Fallén, 1810), *Phyllotelespictipennis* Loew, 1844, *Senotainiaconica* (Fallén, 1810), *Taxigrammahilarella* (Zetterstedt, 1844), *Taxigrammastictica* (Meigen, 1830), *Agriamonachae* (Kramer, 1908), *Nyctialugubris* (Macquart, 1843), Blaesoxipha (Blaesoxipha) aurulenta Rohdendorf, 1937, Blaesoxipha (Blaesoxipha) batilligera Séguy, 1941, Blaesoxipha (Blaesoxipha) plumicornis (Zetterstedt, 1859), Sarcophaga (Helicophagella) okaliana (Lehrer, 1975), Sarcophaga (Heteronychia) amita Rondani, 1860, Sarcophaga (Heteronychia) ancilla Rondani, 1865, Sarcophaga (Heteronychia) pseudobenaci (Baranov, 1942), Sarcophaga (Myorhina) lunigera Böttcher, 1914 and Sarcophaga (Stackelbergeola) mehadiensis Böttcher, 1912. *Taxigrammahilarella*, *Nyctialugubris*, *Agriamonachae*, Blaesoxipha (Blaesoxipha) aurulenta and Sarcophaga (Heteronychia) amita are recorded from Southeast Europe with certainty for the first time. The species Sarcophaga (Sarcophaga) hennigi Lehrer, 1978 is omitted from the list, as previous records from Croatia are shown to be based on an erroneous synonymy with *Sarcophaganovaki* Baranov, 1941 (= Sarcophaga (Sarcophaga) croatica Baranov, 1941). Blaesoxipha (Blaesoxipha) rufipes (Macquart, 1839) could not be confirmed from Croatia and is not included in the checklist. Three new synonymies are proposed: *Golania* Lehrer, 2000 = *Thyrsocnema* Enderlein, 1928, **syn. nov.**, Parasarcophaga (Liosarcophaga) kovatschevitchi Strukan, 1970 = Sarcophaga (Liosarcophaga) marshalli Parker, 1923, **syn. nov.**, and Sarcophagasubvicinassp.novaki Baranov, 1941 = Sarcophaga (Sarcophaga) croatica Baranov, 1941, **syn. nov.** As part of an effort to update the European distributions of all Croatian species, the following new national and regional records are also provided: *Miltogrammabrevipila*, *Miltogrammataeniata* Meigen, 1824 and Sarcophaga (Heteronychia) pandellei (Rohdendorf, 1937) new to Greece; Sarcophaga (Liosarcophaga) harpax Pandellé, 1896 and Sarcophaga (Sarcophaga) croatica new to Italy (respectively mainland and mainland and Sicily); *Miltogrammaiberica* new to Bulgaria and Sardinia; *Pterellaconvergens* (Pandellé, 1895) new to mainland Italy and Sicily; *Nyctialugubris* new to mainland Italy and Sardinia; Blaesoxipha (Blaesoxipha) litoralis (Villeneuve, 1911) new to Sardinia and thus confirmed for Italy; *Apodacraseriemaculata*, *Macronychiastriginervis*, *Protomiltogrammafasciata* (Meigen, 1824) and Blaesoxipha (Blaesoxipha) ungulata (Pandellé, 1896) new to Sardinia and Sicily; *Macronychiadolini* Verves & Khrokalo, 2006, *Macronychiapolyodon* (Meigen, 1824), *Metopiaargyrocephala* (Meigen, 1824), *Senotainiaalbifrons* (Rondani, 1859), *Taxigrammamultipunctata* (Rondani, 1859), *Taxigrammastictica*, Blaesoxipha (Blaesoxipha) unicolor (Villeneuve, 1912) and Sarcophaga (Helicophagella) agnata Rondani, 1860 new to Sardinia; *Metopodiapilicornis* (Pandellé, 1895), *Miltogrammaoestracea*, *Miltogrammarutilans* Meigen, 1824, *Nyctiahalterata* (Panzer, 1798), Blaesoxipha (Blaesoxipha) lapidosa Pape, 1994 and Blaesoxipha (Blaesoxipha) plumicornis new to Sicily.

## Introduction

Sarcophagidae, commonly known as flesh flies because many species feed on the soft tissues of animals (Povolný and Verves 1997), comprise ca 2800 species worldwide (Pape 1996 and unpublished) and are currently divided into three subfamilies: Miltogramminae, Paramacronychiinae and Sarcophaginae (Pape 1996). The flesh fly fauna of Croatia has been poorly studied. The earliest data were published between the mid-19^th^ century and the beginning of the 20^th^ century (Schiner 1862; Brauer and Bergenstamm 1891; Strobl 1893, 1900, 1904; Böttcher 1912, 1913; Langhoffer 1920; Rohdendorf 1937), whereas the first studies more specifically focused on Croatian flesh flies were undertaken before and during World War II by Baranov (1928, 1929, 1930, 1931, 1938, 1939, 1940, 1941a, 1941b, 1942, 1943), who provided information on the general morphology and taxonomy of Croatian Sarcophaginae as well as descriptions and identification keys. Baranov’s work was continued by Strukan (1964, 1967, 1968, 1970), and further data were added after the breakup of Yugoslavia by Povolný and Znojil (1994), Rucner (1994), Pape (1996), Povolný and Znojil (1998, 1999), Pape (2004), Whitmore (2011) and Whitmore et al. (2013). The primary aim of this paper is to summarize all available data on the flesh fly fauna of Croatia, based on a critical review of literature records, on data obtained from recent collecting (2004–2017), and on older specimens in the collections of the Natural History Museum of Denmark (Copenhagen), the National Museum of Natural History, Smithsonian Institution (Washington, D.C.), the Natural History Museum (London) and Museum für Naturkunde (Berlin). We also report 33 new national and regional species records for Bulgaria, Greece, Italy, Sardinia and Sicily.

## Materials and methods

### Study area

**Figure 1.**
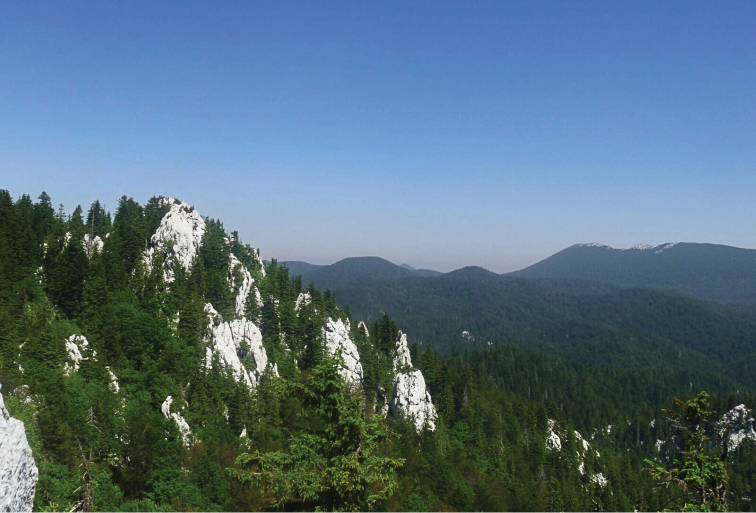
Croatia, Alpine region, Primorsko-goranska Co., 11 km SE Begovo Razdolje, nr Bijele Stijene, 45°13'11"N, 14°58'29"E (photo: E. Buenaventura).

**Figure 2.**
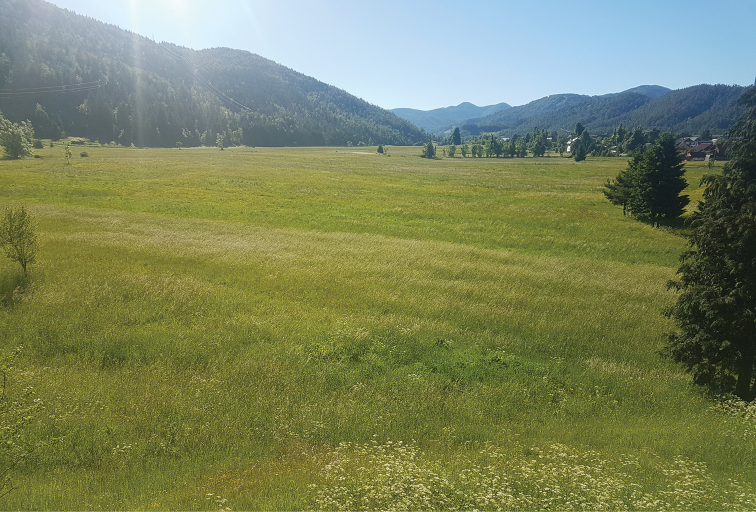
Croatia, Alpine region, Primorsko-goranska Co., Sunger, 45°19'22"N, 14°49'12"E (photo: S. Krčmar).

**Figure 3.**
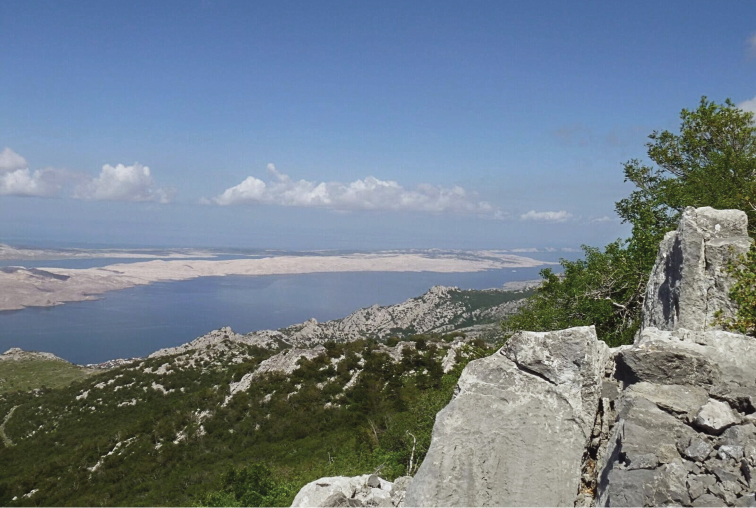
Croatia, Mediterranean region, Ličko-senjska Co., nr Sušanj Cesarički, 44°31'51"N, 15°07'37"E (photo: E. Buenaventura).

**Figure 4.**
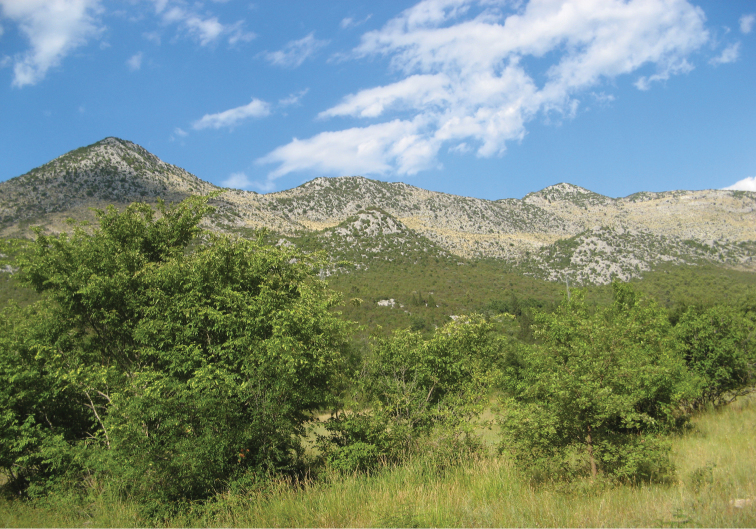
Croatia, Mediterranean region, Dubrovačko-neretvanska Co., nr Vid, 43°04'46"N, 17°37'33"E (photo: S. Krčmar).

**Figure 5.**
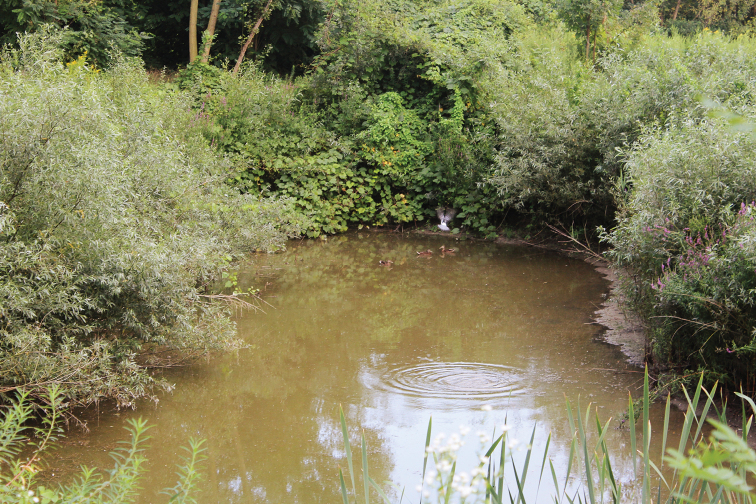
Croatia, Pannonian-Peripannonian region, Osječko-baranjska Co., Zmajevac, 45°48'03"N, 18°48'29"E (photo: S. Krčmar).

**Figure 6.**
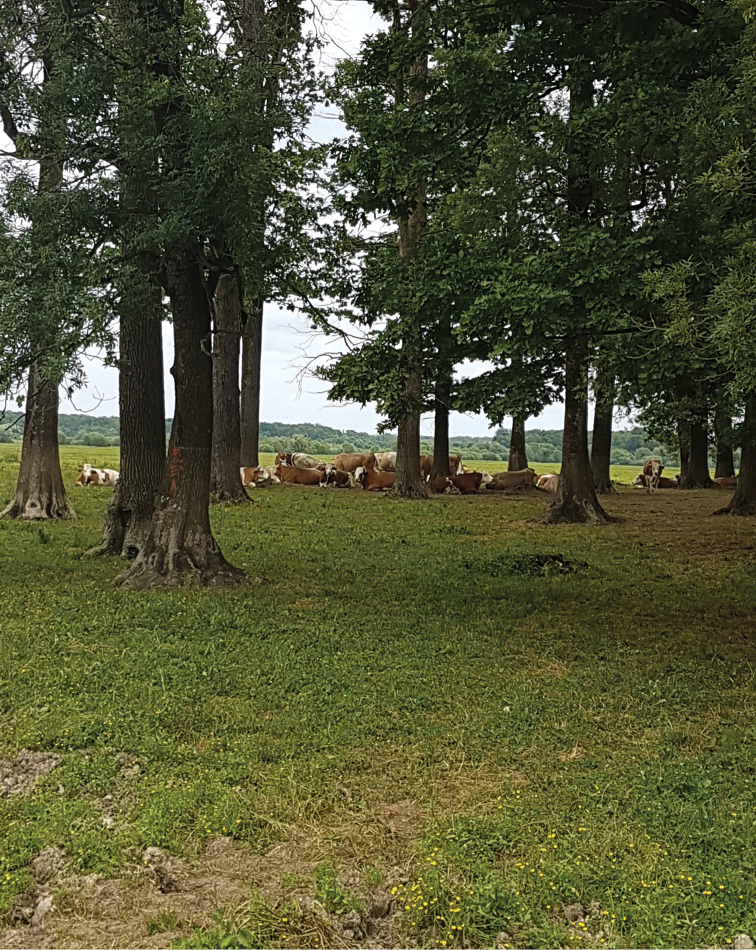
Croatia, Pannonian-Peripannonian region, Sisačko-moslavačka Co., nr Čigoč, 45°24'55"N, 16°37'50"E (photo: S. Krčmar).

Croatia has an area of 56,542 square kilometers and borders with Slovenia, Hungary, Bosnia and Herzegovina, Serbia, Montenegro and Italy. The country is divided into three biogeographical regions: Pannonian-Peripannonian, Alpine, and Mediterranean (Bertić et al. 2001). The Pannonian-Peripannonian region extends between the rivers Drava, Sava and Danube, the Alpine region covers the area of the Dinaric Alps, Gorski Kotar and Lika, whereas the Mediterranean region extends along the Adriatic coast and includes a multitude of islands, cliffs and ridges. The study area includes 38 localities belonging to the Mediterranean, Alpine and Pannonian-Peripannonian regions of Croatia (Figs 1–6, Table 1).

**Table 1.** List of sites sampled for flesh flies (Sarcophagidae) in Croatia between 2004 and 2017.

	Biogeographical region	Locality	Geographic coordinates
1	Pannonian-Peripannonian	Čigoč	45°24'55"N, 16°37'50"E
2		Kamenac	45°45'37"N, 18°42'38"E
3		Orahovica	45°31'44"N, 17°52'49"E
4		Slatinski Drenovac	45°33'01"N, 17°42'27"E
5		Zmajevac	45°48'03"N, 18°48'29"E
6	Alpine	Baške Oštarije	44°31'35"N, 15°10'28"E
7		Begovo Razdolje, nr Bijele Stijene	45°13'11"N, 14°58'29"E
8		Bjelolasica	45°16'24"N, 14°57'40"E
9		Brušane	44°30'05"N, 15°16'08"E
10		Lokve	45°21'30"N, 14°45'03"E
11		Plitvička jezera National Park, Turčić	44°51'55"N, 15°34'55"E
12		Podoštra	44°31'37"N, 15°19'58"E
13		Sertić Poljana	44°55'30"N, 15°34'02"E
14		Skrad	45°25'49"N, 14°54'29"E
15		Sunger	45°19'22"N, 14°49'12"E
16	Mediterranean	Badžula	42°57'53"N, 17°36'41"E
17		Banja	43°03'09"N, 17°30'11"E
18		Biograd	43°55'41"N, 15°24'42"E
19		Blace	43°00'06"N, 17°28'49"E
20		Brač Is., Bol	43°15'44"N, 16°39'15"E
21		Crikvenica	45°10'25"N, 14°41'29"E
22		Desne	43°03'33"N, 17°32'13"E
23		Drivenik	45°14'26"N, 14°38'59"E
24		Hvar Is., Dubovica	43°08'46"N, 16°32'06"E
25		Komin	43°02'30"N, 17°32'09"E
26		Krk Is., Krk	45°01'40"N, 14°34'31"E
27		Krk Is., Punat	45°01'15"N, 14°37'54"E
28		Modro Oko	43°03'25"N, 17°31'05"E
29		Oštrovica	45°22'52"N, 14°51'40"E
30		Pakleni Is., Sveti Klement	43°09'53"N, 16°22'27"E
31		Podrujnica	43°03'34"N, 17°35'31"E
32		Rudelić Draga	44°26'13"N, 15°11'33"E
33		Sušanj Cesarički	44°31'51"N, 15°07'37"E
34		Sveti Juraj	44°55'42"N, 14°55'13"E
35		Tribanj-Krušćica	44°20'59"N, 15°18'59"E
36		Učka Nature Park, Vela Učka	45°18'08"N, 14°11'32"E
37		Vid	43°04'46"N, 17°37'33"E
38		Vriještica	43°04'41"N, 17°35'38"E

### Specimen sampling and identification

The recent sampling effort was carried out between 2004 and 2017. Most flies were collected with hand nets, with additional specimens collected by Malaise traps, plastic bottle traps and multipurpose traps used to collect biting flies. Specimens were preserved in 96% ethanol or killed in ethyl acetate and pinned shortly after sampling. Male terminalia were extended *in loco* when specimens were still fresh, or male and female terminalia were dissected at a later stage following the method described by Richet et al. (2011). Male abdomens were removed and soaked in a 10% KOH solution for 72 hours, immersed in 10% acetic acid for one minute and rinsed in water for another minute before being dehydrated in beech-wood creosote for four hours; the phallus, pre- and postgonites, sternite 5, cerci and surstyli were separated from the rest of the abdomen, placed in a drop of Canada balsam on a microscope slide and covered with a coverslip. Female abdomens were detached, washed in an ethanol solution and left to macerate in a 10% KOH solution for one hour; abdomen tips were then separated by cutting the membrane between the last two tergites, and rinsed in acetic acid and water before being left between slide and coverslip in beech-wood creosote for a period of four hours; they were then mounted in Canada balsam between two coverslips. Identifications were carried out using current keys for Sarcophagidae (Pape 1987; Povolný and Verves 1997; Richet et al. 2011) and descriptions and illustrations in Whitmore (2010, 2011), Whitmore et al. (2013) and Whitmore and Perry (2018).

### Format of checklist

The nomenclature and classification follow the Fauna Europaea database (Pape 2004). For newly collected and museum material, the following information is provided for each record: locality and date of collection, collector(s), number and sex of specimens, and depository; for previously published records, literature sources are listed in chronological order together with locality information, if available. In both sections, main localities (e.g., city, village, island, mountain) are separated by semicolons (viz., Zmajevac; Zagreb; Pag Is.; Medvednica). When two locality names are separated by a comma, the second locality is the exact sampling site and is subordinate to the main locality (e.g., Brač Is., Bol; Medvednica, Medvedgrad; Otočac, Metla; Ston, Broce). Species marked with a black triangle (▲) are recorded for Croatia with certainty for the first time. Species marked with an asterisk (*) are recorded for Croatia based on unverified literature records only. European distributions are derived from Séguy (1941), Pape et al. (1995), Pape (1996), Povolný (1997, 1999), Panu et al. (2000), Pape (2004), Whitmore (2009a, 2009b, 2010), Gaglio et al. (2011), Richet et al. (2011), Whitmore (2011), Richet et al. (2013), Verves and Khrokalo (2014), Whitmore (2016) and Whitmore and Perry (2018), and listed according to the Fauna Europaea format for countries and regions (Pape et al. 2015). Geographic coordinates of recent Croatian sampling localities are given in Table 1. All specimens examined for this study are deposited in the collections of the Natural History Museum of Denmark, Copenhagen, Denmark (NHMD), the National Museum of Natural History, Smithsonian Institution, Washington, D.C., U.S.A. (NMNH), the Department of Biology, Josip Juraj Strossmayer University, Osijek, Croatia (DBUO), the Natural History Museum, London, U.K. (NHMUK), the Museum für Naturkunde, Berlin, Germany (ZMHB), and in the second author’s private collection (DW; currently Stuttgart, Germany). Croatian records published by Strobl (1893, 1900, 1904), Langhoffer (1920), Baranov (1928, 1929, 1930, 1931, 1938, 1939, 1940, 1941a, 1941b, 1942, 1943), Mikačić (1938), Strukan (1964, 1967, 1968, 1970), Povolný (1987), Sisojević et al. (1989), Rucner (1994), Pape (1996), Szpila (2010), Whitmore (2011) and Whitmore et al. (2013) are mostly based on specimens deposited in the following collections: NMNH; NHMD; NHMUK; DW; Moravské Muzeum, Brno, Czech Republic; Naturhistoriska Riksmuseet, Stockholm, Sweden; Centro Nazionale per lo Studio e la Conservazione della Biodiversità Forestale “Bosco Fontana”, Verona, Italy; Department of Ecology and Biogeography, Nicolaus Copernicus University, Toruń, Poland; Croatian Natural History Museum, Zagreb, Croatia; Institute for Biological Research “Siniša Stanković”, Belgrade, Serbia; National Museum of Bosnia and Herzegovina, Sarajevo, Bosnia and Herzegovina; Natural History Museum, Admont, Austria.

## Results

Altogether, 1534 specimens were examined from Croatia and other Mediterranean countries. New locality records are provided for 132 species, including new Croatian records for 105 species. In Croatia, the highest number of species (72) was recorded in the Mediterranean region, followed by the Pannonian-Peripannonian region with 48 species and the Alpine region with 39 species. The most common species in this study was Sarcophaga (Sarcophaga) croatica Baranov, 1941 with 233 specimens from 12 localities. The next most common species were S. (Sarcophaga) lehmanni Müller, 1922 with 172 specimens from 14 localities, S. (Parasarcophaga) albiceps Meigen, 1826 with 70 specimens from 7 localities, and S. (Bellieriomima) subulata Pandellé, 1896 with 40 specimens from 5 localities. The updated checklist of Croatian Sarcophagidae consists of 148 species (two left unnamed): 104 in the subfamily Sarcophaginae, 35 in the subfamily Miltogramminae and 9 in the subfamily Paramacronychiinae. The majority of species belong to the genus *Sarcophaga* Meigen (88), followed by *Blaesoxipha* Loew (15), *Miltogramma* Meigen (11), and *Macronychia* Rondani, *Pterella* Robineau-Desvoidy, *Sphenometopa* Townsend and *Taxigramma* Perris (3 each). The remaining 15 genera are represented by one or two species each. Two species previously recorded from Croatia are omitted from the checklist. *Blaesoxipharufipes* (Macquart, 1839) was listed from Croatia (Pape 1994, 1996; Verves and Khrokalo 2014) based on a misreading of Baranov (1942). Records of Sarcophaga (Sarcophaga) hennigi Lehrer, 1978 are based on an erroneous synonymy, as discussed below under S. (S.) croatica. As part of an effort to update the distributions of all Croatian species, we here firstly report the occurrences of one species for Bulgaria, three species for Greece (Corfu), two species for Italy, 15 species for Sardinia and 12 species for Sicily (for details, see Abstract and Remarks under individual species).

### Checklist

#### Subfamily Miltogramminae Lioy, 1864

##### 1. *Amobiapelopei* (Rondani, 1859) (▲)

Records: Gračac, 6.X.1929, N. Baranov leg. (1♂) (NMNH); same locality, 7.X.1929, N. Baranov leg. (1♂) (NMNH); same locality, 10.X.1929, N. Baranov leg. (1♂) (NMNH); Mraclin, 22.VI.1930, N. Baranov leg. (1♂) (NMNH); Pag Is., VIII.1933, N. Baranov leg. (1♂) (NMNH); same locality, VIII.1935, N. Baranov leg. (2♂) (NMNH).

European distribution: Austria, Croatia, Czech Republic, France (mainland), Germany, Hungary, Italy (mainland), Poland, Russia (South European Territory), Switzerland, Ukraine.

##### 2. *Amobiasignata* (Meigen, 1824)

New records: Mraclin, 19.VIII.1928, N. Baranov leg. (1♂) (NMNH); Brač Is., Bol, 5.VIII.2004, D. Whitmore leg. (1♀) (DW); Gornje Igrane, 18.V.2012, C. Lange, J. Ziegler leg. (1♂) (ZMHB).

Literature records: Split (Strobl 1900); Pape (2004); Slano, nr Dubrovnik (Szpila 2010).

European distribution: Albania, Austria, Belgium, Bulgaria, Croatia, Cyprus, Czech Republic, Denmark (mainland), France (mainland, Corsica), Germany, Greece (mainland), Hungary, Italy (mainland, Sardinia, Sicily), Lithuania, Malta, Moldova, Poland, Romania, Russia (Central European Territory, South European Territory), Serbia, Slovakia, Slovenia, Spain (mainland, Canary Is.), Sweden, Switzerland, The Netherlands, Ukraine, United Kingdom.

##### 3. *Apodacraseriemaculata* Macquart, 1854 (▲)

Records: Đurđenovac, 20.VII.1901, N. Baranov leg. (1♂) (NMNH).

European distribution: Austria, Croatia, France (mainland, Corsica), Italy (mainland, Sardinia, Sicily), Romania, Russia (South European Territory), Spain (mainland), Ukraine.

Remarks: We here record this species from Sardinia (Sassari, Stintino, 9.VII.2004, D. Birtele leg., 1♀, DW; Arbus, Piscinas, 39°32'25.62"N, 8°27'7.88"E, 25.V.2006, D. Whitmore leg., 1♀, DW; same as previous except 14.VII.2006, 2♀, 3♂, DW; Domusnovas, Valle Oridda, 39°24'32.16"N, 8°36'58.94"E, 592 m, 11.VII.2006, D. Whitmore leg., 1♂, DW) and Sicily (Agrigento, Torre Salsa, 22.V.2004, D. Whitmore leg., 1♀, 2♂, DW) for the first time.

##### 4. *Craticulinatabaniformis* (Fabricius, 1805) (▲)

Records: Pag Is., VIII.1934, N. Baranov leg. (2♂) (NMNH).

European distribution: Austria, Croatia, France (mainland, Corsica), Hungary, Italy (mainland, Sardinia, Sicily), Malta, Romania, Russia (South European Territory), Spain (mainland), Ukraine.

##### 5. *Macronychiadolini* Verves & Khrokalo, 2006

New records: Baške Oštarije, 13.VI.2012, E. Buenaventura, T. Pape, D. Whitmore leg. (2♂) (NHMD).

Literature records: Verves and Khrokalo (2006).

European distribution: Croatia, Czech Republic, France (mainland), Italy (mainland, Sardinia), Poland, Russia (South European Territory), Sweden, Switzerland, The Netherlands, Ukraine, United Kingdom.

Remarks: We here record this species from Sardinia for the first time (Domusnovas, Valle Oridda, 39°24'32.16"N, 8°36'58.94"E, 592 m, 11.VII.2006, D. Whitmore leg., 1♂, DW; Domusnovas, Bega d’Aleni, 39°24'1.76"N, 8°37'33.66"E, 621 m, 17.VII.2006, D. Birtele leg., 1♀, DW).

##### 6. *Macronychiapolyodon* (Meigen, 1824)

New records: Krapina, VIII.1927, N. Baranov leg. (1♀) (NMNH); Samobor, 25.VI.1930, N. Baranov leg. (1♂) (NMNH); Baške Oštarije, 13.VI.2012, E. Buenaventura, T. Pape, D. Whitmore leg. (1♂) (NHMD); Rudelić Draga, 14.VI.2012, E. Buenaventura, T. Pape, D. Whitmore leg. (1♂) (NHMD); Podoštra, 15.VI.2012, E. Buenaventura, T. Pape, D. Whitmore leg. (1♂) (NHMD).

Literature records: Krapina; Mraclin; Pag Is.; Samobor (Baranov 1938); Verves and Khrokalo (2006).

European distribution: Andorra, Austria, Belgium, Bulgaria, Croatia, Czech Republic, Estonia, Finland, France (mainland), Germany, Hungary, Italy (mainland, Sardinia), Malta, Moldova, Norway (mainland), Poland, Romania, Russia (Central European Territory, South European Territory), Slovakia, Spain (mainland), Sweden, Switzerland, The Netherlands, Ukraine, United Kingdom.

Remarks: We here record this species from Sardinia for the first time (Iglesias, Monti Marganai, 480 m, 7–11.VI.2004, P. Cerretti et al. leg., 1♀, 4♂, DW).

##### 7. *Macronychiastriginervis* (Zetterstedt, 1838) (▲)

Records: Mraclin, 9.VIII.1928, N. Baranov leg. (3♀) (NMNH); same locality, 6.VII.1929, N. Baranov leg. (1♀) (NMNH); same locality, 26.VI.1930, N. Baranov leg. (1♀) (NMNH).

European distribution: Austria, Croatia, Czech Republic, Denmark (mainland), Estonia, Finland, France (mainland), Germany, Hungary, Italy (mainland, Sardinia, Sicily), Portugal (Madeira Is.), Moldova, Norway (mainland), Poland, Romania, Russia (Central European Territory, South European Territory), Slovakia, Sweden, Switzerland, The Netherlands, Ukraine, United Kingdom.

Remarks: We here record this species from Sardinia (Domusnovas, Bega d’Aleni, 621 m, 39°24'1.76"N, 8°37'33.66"E, 15–17.VII.2006, D. Whitmore leg., 2♀, 1♂, DW; Iglesias, Conca Margiani, 39°21'40.76"N, 8°33'44.05"E, 750 m, D. Whitmore leg., 2♀, DW) and Sicily (Palermo, Bosco della Ficuzza, 27.VI.2005, P. Cerretti leg., 1♀, DW; Palermo, Corleone, nr Bivio Ponte Casale, 37°50'55.27"N, 13°20'9.35"E, 476 m, 27–30.VI.2005, D. Whitmore leg., 2♂, DW; Palermo, Parco delle Madonie, Petralia, Gorgo Nero, 1157 m, 29.VI.2005, D. Whitmore leg., 1♀, DW) for the first time.

##### 8. *Metopiaargyrocephala* (Meigen, 1824)

New records: Krapina, 28.VII.1929, N. Baranov leg. (1♀) (NMNH); Mraclin, 9.VII.1928, N. Baranov leg. (1♂) (NMNH); Zagreb, Podsused, 3.V.1931, N. Baranov leg. (1♀) (NMNH); Samobor, 22.IX.1929, N. Baranov leg. (1♀) (NMNH); Zagreb, 2.VII.1928, N. Baranov leg. (1♂) (NMNH); same locality, 16.VIII.1929, N. Baranov leg. (1♂) (NMNH); Vis Is., Komiža, 24.VI.1973, M. Chvála leg. (1♀) (NHMUK); Sveti Juraj, 12.VI.2012, E. Buenaventura, T. Pape, D. Whitmore leg. (1♂) (NHMD).

Literature records: Delnice; Lokve; Plitvice (Langhoffer 1920, as *leucocephala* Rossi); Baranov (1928, as *leucocephala*).

European distribution: Andorra, Austria, Belarus, Belgium, Bulgaria, Croatia, Cyprus, Czech Republic, Denmark (mainland), Estonia, Finland, France (mainland, Corsica), Germany, Greece (mainland), Hungary, Italy (mainland, Sardinia, Sicily), Latvia, Lithuania, Moldova, Norway (mainland), Poland, Romania, Russia (Central European Territory, North European Territory, South European Territory), Serbia, Slovakia, Spain (mainland), Sweden, Switzerland, The Netherlands, Ukraine, United Kingdom.

Remarks: We here record this species from Sardinia for the first time (Buggerru, Portixeddu, 14.VI.2004, P. Cerretti et al. leg., 1♀, DW; Villacidro, Canale Monincu, 39°25'10.01"N, 8°37'40.61"E, 450 m, 21.V.2006, D. Whitmore leg., 3♂, DW; Domusnovas, Valle Oridda, 39°24'32.16"N, 8°36'58.94"E, 592 m, 24.V.2006, D. Whitmore leg., 3♂, DW; Villacidro, S’Acqua Frischedda, 39°24'51.74"N, 8°37'58.13"E, 390 m, D. Whitmore leg., 1♀, DW; Arbus, Piscinas, 39°32'25.62"N, 8°27'7.88"E, 25.V.2006, D. Whitmore leg., 1♂, DW; Villacidro, Lago di Montimannu, 39°25'5.85"N, 8°41'58.18"E, 256 m, 10.VII.2006, D. Whitmore leg., 2♂, DW; Domusnovas, Punta Planotzara, 39°21'16.71"N, 8°35'59.09"E, 360 m, 13.VII.2006, D. Whitmore leg., 1♂, DW; Siniscola, Santa Lucia, 26.VII.2009, D. Birtele leg., 1♀, DW).

##### 9. *Metopiacampestris* (Fallén, 1810) (▲)

Records: Krapina, 2.VIII.1929, N. Baranov leg. (1♂) (NMNH).

European distribution: Albania, Andorra, Austria, Belarus, Belgium, Bulgaria, Croatia, Czech Republic, Denmark (mainland), Estonia, Finland, France (mainland), Germany, Hungary, Ireland, Italy (mainland), Latvia, Moldova, Norway (mainland), Poland, Romania, Russia (Central European Territory, North European Territory, South European Territory), Serbia, Slovakia, Spain (mainland), Sweden, Switzerland, The Netherlands, Ukraine, United Kingdom.

##### 10. *Metopodiapilicornis* (Pandellé, 1895)

Literature records: Pape (1996, 2004); Slano, nr Dubrovnik (Szpila 2010).

European distribution: Croatia, Cyprus, Czech Republic, France (mainland), Germany, Hungary, Italy (mainland, Sardinia, Sicily), Russia (South European Territory), Spain (mainland).

Remarks: We here record this species from Sicily for the first time (Palermo, Bosco della Ficuzza, Cima Cucco, 37°52'11.41"N, 13°24'34.56"E, 995 m, D. Whitmore leg., 5♂, DW).

##### 11. *Miltogrammabrevipila* Villeneuve, 1911 (▲)

Records: Pag Is., 30.IX.1932, N. Baranov leg. (1♀) (NMNH).

European distribution: Austria, Croatia, Czech Republic, France (mainland, Corsica), Greece (mainland), Italy (mainland, Sicily), Norway (mainland), Romania, Slovakia, Russia (South European Territory), Spain (mainland), Sweden, Switzerland, Ukraine.

Remarks: We here record this species from Greece for the first time (Corfu, Agios Gordios, 39°33'2.39"N, 19°50'51.06"E, 7.IX.2016, D. Whitmore leg., 2♂, DW).

##### 12. *Miltogrammacontarinii* Rondani, 1859 (*)

Literature records: Senj (Langhoffer 1920).

Distribution: Croatia, France (mainland), Italy (mainland).

Remarks: Unverified record. Langhoffer (1920) published a list of 1323 species in 50 Diptera families from Croatia, but these data are partly outdated (Britvec 2000) and many species may have been misidentified. *Miltogrammacontarinii* is a rare species so far recorded with certainty only from Italy and France.

##### 13. *Miltogrammagermari* Meigen, 1824

New records: Krapina, 10.VII.1923, N. Baranov leg. (1♀, 1♂) (NMNH); Mraclin, 2.VII.1928, N. Baranov leg. (1♂) (NMNH); same locality, 9.VIII.1928, N. Baranov leg. (1♀) (NMNH).

Literature records: Đurđevac (Langhoffer 1920); Baranov (1928).

European distribution: Austria, Belgium, Bulgaria, Croatia, Czech Republic, Denmark (mainland), Finland, France (mainland), Germany, Hungary, Italy (mainland), Latvia, Moldova, Poland, Romania, Russia (Central European Territory, South European Territory), Slovakia, Sweden, Switzerland, The Netherlands, Ukraine, United Kingdom.

##### 14. *Miltogrammaiberica* Villeneuve, 1912 (▲)

Records: Krapina, 29.VIII.1910, N. Baranov leg. (1♂) (NMNH); same locality, 4.IX.1912, N. Baranov leg. (1♂) (NMNH); Mraclin, 19.VII.1933, N. Baranov leg. (1♀) (NMNH).

European distribution: Austria, Bulgaria, Croatia, Finland, Hungary, Italy (mainland, Sardinia), Russia (Central European Territory, South European Territory), Spain (mainland), Ukraine.

Remarks: We here record this species from Bulgaria (Burgas, Tsarevo, nr Sinemorets, 42°3'1.68"N, 27°59'3.32"E, 28.V.2018, D. Whitmore leg., 1♀, DW) and Sardinia (Palau, Porto Rafael, 1–15.IX.1997, P. Cerretti leg., 1♀, 2♂, DW; Iglesias, Monti Marganai, 500 m, 2.IX.2003, P. Cerretti leg., 1♂, DW; Domusnovas, Grotta San Giovanni, 12.VI.2004, D. Birtele et al. leg., 1♂, DW; Iglesias, Monti Marganai, 480 m, 22.IX.2004, D. Birtele et al. leg., 1♀, DW; Domusnovas, Sa Duchessa, 308 m, 25.IX.2004, P. Cerretti et al. leg., 2♀, DW; Domusnovas, Bega d’Aleni, 39°24'1.76"N, 8°37'33.66"E, 621 m, 17.VII.2006, D. Whitmore leg., 3♀, 1♂, DW; same as previous except P. Cerretti leg., 1♂, DW; same as previous except D. Birtele leg., 2♂, DW; Iglesias, Vecchia Cantoniera Marganai, 39°20'26.52"N, 8°33'43.90"E, 491 m, 23.V.2006, D. Whitmore leg., 1♂, DW; Domusnovas, Rio Sa Duchessa, 39°21'41.85"N, 8°36'48.46"E, 270 m, 12.VII.2006, D. Whitmore leg., 2♂, DW; Domusnovas, Sa Duchessa, 39°22'27.18"N, 8°35'36.74"E, 371 m, 12.VI.2006, P. Cerretti leg., 1♀, DW; Domusnovas, Valle Oridda, 39°24'32.16"N, 8°36'58.94"E, 592 m, 15.VII.2006, D. Whitmore leg., 1♂, DW; Tortolì, Lido di Orri, 2.VIII.2009. D. Birtele leg., 1♀, DW) for the first time.

##### 15. *Miltogrammamurina* Meigen, 1824

New records: Brač Is., Bol, 4.VIII.2004, D. Whitmore leg. (1♀) (DW); Hvar Is., Dubovica, 12.VIII.2004, D. Whitmore leg. (1♀) (DW).

Literature records: Bakar (Langhoffer 1920); Baranov (1928); Slano, nr Dubrovnik (Szpila 2010).

European distribution: Belgium, Croatia, Czech Republic, Germany, Greece (mainland), Hungary, Italy (mainland, Sardinia, Sicily), Malta, Poland, Romania, Slovakia, Ukraine.

##### 16. *Miltogrammaoestracea* (Fallén, 1820) (▲)

Records: Srijem, 8.VII.1930, N. Baranov leg. (1♂) (NMNH).

European distribution: Albania, Austria, Belarus, Belgium, Czech Republic, Denmark (mainland), Estonia, Finland, France (mainland), Germany, Hungary, Italy (mainland, Sicily), Latvia, Moldova, Poland, Romania, Russia (Central European Territory, South European Territory), Serbia, Slovakia, Spain (mainland), Sweden, Ukraine.

Remarks: We here record this species from Sicily for the first time (Trapani, Oasi dello Zingaro, 9.V.2004, D. Whitmore leg., 3♂, DW; Palermo, Corleone, 476 m, 27.VI.2005, D. Whitmore leg., 1♂, DW).

##### 17. *Miltogrammapunctata* Meigen, 1824 (▲)

Records: Krapina, 13.VIII.1912, N. Baranov leg. (1♂) (NMNH); Orebić, VI.1927, Ziegenhagen leg. (2♂) (ZMHB).

European distribution: Austria, Belarus, Belgium, Bulgaria, Croatia, Cyprus, Czech Republic, Denmark (mainland), Estonia, Finland, France (mainland, Corsica), Germany, Greece (mainland), Hungary, Ireland, Italy (mainland, Sardinia, Sicily), Moldova, Norway (mainland), Poland, Romania, Russia (Central European Territory, South European Territory), Slovakia, Spain (mainland, Canary Is.), Sweden, Switzerland, The Netherlands, Ukraine, United Kingdom.

##### 18. *Miltogrammaruficornis* Meigen, 1824 (*)

Literature records: Rijeka; Hvar (Strobl 1893); Krka Falls (Strobl 1900); Hvar (Strobl 1904).

European distribution: Albania, Austria, Belgium, Croatia, France (mainland, Corsica), Germany, Hungary, Italy (mainland), Poland, Spain (mainland).

Remarks: Unverified records. Species of *Miltogramma* can easily be misidentified, and misidentifications in older publications cannot be excluded.

##### 19. *Miltogrammarutilans* Meigen, 1824

New records: Pag Is., VIII.1933, N. Baranov leg. (4♂) (NMNH); same locality, 26.VIII.1933, N. Baranov leg. (1♂) (NMNH); same locality, VIII.1935, N. Baranov leg. (3♂) (NMNH).

Literature records: Pag Is., Kolansko Blato (Baranov 1938).

European distribution: Austria, Croatia, Czech Republic, France (mainland, Corsica), Germany, Hungary, Italy (mainland, Sardinia, Sicily), Malta, Poland, Russia (Central European Territory, South European Territory), Slovakia, Ukraine.

Remarks: We here record this species from Sicily for the first time (Sicily, Corleone, nr Bivio Ponte Casale, 37°50'55.27"N, 13°20'9.35"E, 476 m, 30.VI.2005, D. Whitmore leg., 2♀, DW).

##### 20. *Miltogrammataeniata* Meigen, 1824

New records: Brač Is., Bol, 5.VIII.2004, D. Whitmore leg. (1♀) (DW); Tučepi, 16.V.2012, C. Lange, J. Ziegler leg. (1♂) (ZMHB); Sveti Juraj, 12.VI.2012, E. Buenaventura, T. Pape, D. Whitmore leg. (1♂) (NHMD).

Literature records: Rijeka (Strobl 1893, as *pilimana* Rondani); Krka Falls (Strobl 1900, as *pilimana*); Plešce; Selce; Senj (Langhoffer 1920); Baranov (1928); Jakišnica; Krapina; Samobor (Baranov 1938); Paklenica, Velebit Mts; Brač Is., Milna; Vozilići (Sisojević et al. 1989); Slano, nr Dubrovnik (Szpila 2010).

European distribution: Albania, Bulgaria, Croatia, Czech Republic, France (mainland, Corsica), Germany, Greece (mainland), Hungary, Italy (mainland), Poland, Serbia, Slovakia, Spain (mainland), Switzerland, Ukraine.

Remarks: We here record this species from Greece for the first time (Corfu, Agios Gordios, 39°33'2.39"N, 19°50'51.06"E, 7.IX.2016, D. Whitmore leg., 3♂, DW).

##### 21. *Miltogrammatestaceifrons* (von Roser, 1840)

New records: Krapina, 13.VII.1910, N. Baranov leg. (1♂) (NMNH); same locality, 3.VI.1923, N. Baranov leg. (2♂) (NMNH); same locality, 6.VII.1930, N. Baranov leg. (2♂) (NMNH); Zagreb, 4.VII.1929, N. Baranov leg. (1♂) (NMNH).

Literature records: Starigrad (Langhoffer 1920, as *pilitarsis* Rondani).

European distribution: Austria, Croatia, Belarus, Czech Republic, France (mainland), Germany, Hungary, Italy (mainland), Lithuania, Moldova, Poland, Russia (Central European Territory, South European Territory), Slovakia, Spain (mainland), Sweden, Switzerland, The Netherlands, Ukraine.

##### 22. *Oebalia cylindrica* (Fallén, 1810) (▲)

Records: Krapina, N. Baranov leg. (1♂) (NMNH).

European distribution: Andorra, Austria, Belgium, Croatia, Czech Republic, Denmark (mainland), Finland, France (mainland), Germany, Hungary, Italy (mainland), Norway (mainland), Poland, Russia (Central European Territory, South European Territory), Sweden, Switzerland, The Netherlands, Ukraine, United Kingdom.

##### 23. *Phyllotelespictipennis* Loew, 1844 (▲)

Records: Susedgrad, 11.VII.1931, N. Baranov leg. (1♂) (NMNH); same locality, 28.VII.1931, N. Baranov leg. (2♂) (NMNH).

European distribution: Albania, Austria, Bulgaria, Croatia, Cyprus, Czech Republic, France (mainland, Corsica), Germany, Greece (mainland), Hungary, Italy (mainland, Sicily), Macedonia, Poland, Russia (Central European Territory, North European Territory, South European Territory), Serbia, Slovakia, Spain (mainland), Switzerland, Ukraine.

##### 24. *Protomiltogrammafasciata* (Meigen, 1824)

Literature records: Senj (Langhoffer 1920); Baranov (1928); Pape (2004).

European distribution: Austria, Croatia, Czech Republic, France (mainland, Corsica), Germany, Hungary, Italy (mainland, Sardinia, Sicily), Poland, Russia (Central European Territory, South European Territory), Slovakia, Spain (mainland, Canary Is.), Ukraine.

Remarks: We here record this species from Sardinia (Palau, Porto Rafael, 1–15.IX.1997, P. Cerretti leg., 1♀, 2♂, DW; Stintino, Cala Coscia di Donna, 9.VII.2004, D. Birtele leg., 1♂, DW) and Sicily (Palermo, Bosco della Ficuzza, 30.VII.2003, P. Cerretti leg., 4♀, DW) for the first time.

##### 25. *Pterellaconvergens* (Pandellé, 1895)

Literature records: Pag Is.; Samobor (Baranov 1938).

European distribution: Croatia, France (mainland, Corsica), Germany, Greece (mainland), Italy (mainland, Sardinia, Sicily), Poland, Ukraine.

Remarks: We here record this species from mainland Italy (Latium, Rome, Tivoli, Colle Vescovo, 448 m, 18.IX.1999, M. Mei leg., 2♂, M. Mei collection) and Sicily (Palermo, nr Corleone, 27.VI.2005, D. Whitmore leg., 1♀, DW) for the first time. It had previously been recorded from Sardinia by Venturi (1966).

##### 26. *Pterellagrisea* (Meigen, 1824)

New records: Pag Is., 27.VIII.1933, N. Baranov leg. (1♂) (NMNH); same locality, II.1935, N. Baranov leg. (1♂) (NMNH); same locality, VIII.1935, N. Baranov leg. (4♂) (NMNH); same locality, 19.VIII.1935, N. Baranov leg. (2♀) (NMNH); Zagreb, 28.VII.1929, N. Baranov leg. (1♀) (NMNH).

Literature records: Rijeka; Hvar (Strobl 1893, as *intricata* Meigen); Hvar (Strobl 1900, as *intricata*).

European distribution: Austria, Bulgaria, Croatia, Czech Republic, Denmark (mainland), Estonia, Finland, France (mainland), Germany, Hungary, Italy (mainland, Sicily), Latvia, Lithuania, Moldova, Poland, Russia (Central European Territory, South European Territory), Slovakia, Serbia, Sweden, Switzerland, The Netherlands, Ukraine, United Kingdom.

##### 27. *Pterellamelanura* (Meigen, 1824)

New records: Sušanj Cesarički, 13.VI.2012, E. Buenaventura, D. Whitmore, T. Pape leg. (1♀) (NHMD); Tribanj-Kruščica, Ljubotić, 14.VI.2012, E. Buenaventura, T. Pape, D. Whitmore leg. (1♀) (NHMD).

Literature records: Zadar (Strobl 1904); Pape (2004).

European distribution: Albania, Austria, Croatia, Czech Republic, Estonia, France (mainland, Corsica), Germany, Hungary, Italy (mainland, Sardinia, Sicily), Macedonia, Poland, Romania, Russia (Central European Territory, South European Territory), Slovakia, Slovenia, Spain (mainland), Ukraine.

##### 28. *Senotainiaalbifrons* (Rondani, 1859)

New records: Hvar Is., Dubovica, 12.VIII.2004, D. Whitmore leg. (3♀, 2♂) (DW); Krk Is., Glavotok, 12–15.VII.2003, T. Pape leg. (1♂) (NHMD).

Literature records: Hvar; Split (Strobl 1900); Zadar (Strobl 1904); Pape (2004).

European distribution: Austria, Bulgaria, Croatia, Cyprus, Czech Republic, France (mainland, Corsica), Germany, Greece (mainland), Hungary, Italy (mainland, Sardinia, Sicily), Lithuania, Moldova, Poland, Romania, Russia (Central European Territory, South European Territory), Slovakia, Spain (mainland), Switzerland, The Netherlands, Ukraine.

Remarks: We here record this species from Sardinia for the first time (Iglesias, Monti Marganai, 500 m, 1–2.IX.2003, P. Cerretti leg., 2♂, DW; Domusnovas, Sa Duchessa, 308 m, 25.IX.2004, P. Cerretti et al. leg., 1♀, DW; Villacidro, Rio Cannisoni, 39°25'2.26"N, 8°38'1.09"E, 463 m, 21.V.2006, D. Whitmore leg., 1♀, DW; same as previous except 11.VII.2006, 1♂, DW; Arbus, Piscinas, 39°32'25.62"N, 8°27'7.88"E, 25.V.2006, D. Whitmore leg., 2♀, 3♂, DW; same as previous except 14.VII.2006, 7♂, DW; Villacidro, Lago di Montimannu, 39°25'5.85"N, 8°41'58.18"E, 256 m, 10.VII.2006, D. Whitmore leg., 2♀, 3♂, DW; Domusnovas, Valle Oridda, 39°24'32.16"N, 8°36'58.94"E, 592 m, 11–17.VII.2006, D. Whitmore leg., 3♀, 6♂, DW; Domusnovas, Rio Sa Duchessa, 39°21'41.85"N, 8°36'48.46"E, 270 m, 12–18.VII.2006, D. Whitmore leg., 5♀, 11♂, DW; Iglesias, ex Colonia Beneck, 39°20'51.32"N, 8°33'48.71"E, 636 m, 13.VII.2006, D. Whitmore leg., 1♂, DW; Domusnovas, Bega d’Aleni, 39°24'1.76"N, 8°37'33.66"E, 621 m, 17.VII.2006, D. Whitmore leg., 1♀, 1♂, DW; Domusnovas, Sa Duchessa, 39°22'27.18"N, 8°35'36.74"E, 371 m, 7.IX.2006, D. Birtele leg., 1♂, DW).

##### 29. *Senotainiaconica* (Fallén, 1810) (▲)

Records: Zagreb, 21.VII.1930, N. Baranov leg. (1♂) (NMNH).

European distribution: Austria, Belarus, Belgium, Bulgaria, Croatia, Czech Republic, Denmark (mainland), Estonia, Finland, France (mainland), Germany, Hungary, Italy (mainland), Moldova, Norway (mainland), Poland, Romania, Russia (Central European Territory, South European Territory), Slovakia, Sweden, Switzerland, The Netherlands, Ukraine, United Kingdom.

##### 30. *Sphenometopamannii* (Brauer & Bergenstamm, 1891)

Literature records: Rijeka (Brauer and Bergenstamm 1891); Pape (2004).

European distribution: Croatia.

##### 31. *Sphenometopasteinii* (Schiner, 1862)

Literature records: Dubrovnik (Schiner 1862; Strobl 1900); Pape (1996, 2004).

European distribution: Croatia, Cyprus, Greece (mainland).

##### 32. *Sphenometopavariegata* (Stein, 1924)

New records: Hvar Is., nr Jelsa, 5–8.VII.2003, T. Pape leg. (2♂, 2♀) (NHMD); Rudelić Draga, 14.VI.2012, E. Buenaventura, T. Pape, D. Whitmore leg. (1♂, 1♀) (NHMD).

Literature records: Dalmatia (Verves 1986); Pape (1996, 2004).

Distribution: Croatia, France (mainland).

Remarks: This species was probably described from Croatia (Dalmatia), even though the type locality was originally given by Stein (1924) as “Europe” (see Pape 1996). Pape (2004) listed it for Croatia but considered its presence in France as doubtful.

##### 33. *Taxigrammahilarella* (Zetterstedt, 1844) (▲)

Records: Samobor, 21.VII.1929, N. Baranov leg. (1♂) (NMNH); Zagreb, 17.VIII.1930, N. Baranov leg. (2♂) (NMNH); Sveti Juraj, 12.VI.2012, E. Buenaventura, T. Pape, D. Whitmore leg. (1♂) (NHMD); Sušanj Cesarički, 13.VI.2012, E. Buenaventura, D. Whitmore, T. Pape leg. (1♀) (NHMD); Baške Oštarije, 13.VI.2012, E. Buenaventura, T. Pape, D. Whitmore leg. (1♂, 1♀) (NHMD); Rudelić Draga, 14.VI.2012, E. Buenaventura, T. Pape, D. Whitmore leg. (1♂) (NHMD); Brušane, 15.VI.2012, E. Buenaventura, T. Pape, D. Whitmore leg. (2♂) (NHMD); Podoštra, 15.VI.2012, E. Buenaventura, T. Pape, D. Whitmore leg. (1♂) (NHMD).

European distribution: Austria, Belgium, Canary Is., Croatia, Czech Republic, Denmark (mainland), Estonia, Finland, France (mainland), Germany, Hungary, Italy (mainland), Lithuania, Moldova, Poland, Russia (Central European Territory, South European Territory), Slovakia, Sweden, Switzerland, The Netherlands, Ukraine, United Kingdom.

##### 34. *Taxigrammamultipunctata* (Rondani, 1859)

Literature records: Pape (2004).

European distribution: Austria, Bulgaria, Belarus, Croatia, Cyprus, France (mainland, Corsica), Germany, Greece (mainland), Hungary, Italy (mainland, Sardinia, Sicily), Macedonia, Malta, Russia (South European Territory), Serbia, Spain (mainland, Canary Is.), Ukraine.

Remarks: We here record this species from Sardinia for the first time (Iglesias, Monti Marganai, 500 m, 2.IX.2003, P. Cerretti leg., 2♂, DW; Domusnovas, nr Agriturismo Perda Niedda, 350 m, 8.VI.2004, P. Cerretti et al. leg., 1♂, DW; nr Iglesias, 550 m, 26.IX.2004, D. Birtele leg., 1♂, DW; Villacidro, Rio Cannisoni, 39°24'51.10"N, 8°38'0.98"E, 401 m, 19.V.2006, D. Whitmore leg., 2♂, DW; Villacidro, Canale Monincu, 39°25'10.01"N, 8°37'40.61"E, 450 m, 21.V.2006, D. Whitmore leg., 1♂, DW; Villacidro, Lago di Montimannu, 39°25'5.85"N, 8°41'58.18"E, 256 m, 10.VII.2006, D. Whitmore leg., 1♂, DW; 39°24'37.47"N, 8°38'27.65"E, 390 m, 11.VII.2006, D. Whitmore leg., 1♂, DW; Domusnovas, Sa Duchessa, 39°22'27.18"N, 8°35'36.74"E, 371 m, 12.VI.2006, D. Whitmore leg., 1♂, DW; Iglesias, ex Colonia Beneck, 39°20'51.32"N, 8°33'48.71"E, 636 m, 13.VII.2006, D. Whitmore leg., 1♂, DW; Villacidro, Cuccuruneddu, 39°22'8.68"N, 8°40'45.65"E, 708 m, 13.VII.2006, D. Whitmore leg., 1♀, DW; Buggerru, Rio Mannu, 14.VII.2006, D. Whitmore leg., 2♀, DW; Domusnovas, Valle Oridda, 39°24'32.16"N, 8°36'58.94"E, 592 m, 11.VII.2006, D. Whitmore leg., 1♀, 3♂, DW).

NB: These Sardinian specimens do not all fall within the range of variation of *T.multipunctata* defined by Richet et al. (2013), particularly with regard to setation of the parafacialia. Further studies are required to determine whether *T.multipunctata sensu auct.* is a morphologically variable species or a species complex.

##### 35. *Taxigrammastictica* (Meigen, 1830) (▲)

Records: Otočac, VIII.1931, N. Baranov leg. (1♂) (NMNH).

European distribution: Andorra, Austria, Bulgaria, Croatia, Czech Republic, Denmark (mainland), France (mainland), Germany, Hungary, Italy (mainland, Sardinia), Poland, Russia (Central European Territory, South European Territory), Serbia, Sweden, Switzerland, The Netherlands, Ukraine.

Remarks: We here record this species from Sardinia for the first time (Domusnovas, Rio Sa Duchessa, 39°21'41.85"N, 8°36'48.46"E, 270 m, 12.VII.2006, D. Whitmore leg., 1♂, DW).

#### Subfamily Paramacronychiinae Brauer & Bergenstamm, 1889

##### 36. *Agriaaffinis* (Fallén, 1817)

Literature records: Sisojević et al. (1989, as *punctata* Robineau-Desvoidy); Pape (2004).

European distribution: Andorra, Belarus, Belgium, Bulgaria, Croatia, Czech Republic, Denmark (mainland), Estonia, Finland, France (mainland), Germany, Hungary, Italy (mainland, Sardinia), Moldova, Poland, Romania, Russia (Central European Territory, South European Territory), Slovakia, Spain (mainland), Sweden, Switzerland, The Netherlands, Ukraine, United Kingdom.

##### 37. *Agriamonachae* (Kramer, 1908) (▲)

Records: Baške Oštarije, 13.VI.2012, E. Buenaventura, T. Pape, D. Whitmore leg. (1♂) (NHMD).

European distribution: Belarus, Croatia, Czech Republic, Germany, Poland, Russia (Central European Territory), Slovakia, Switzerland, Ukraine.

##### 38. *Brachicomadevia* (Fallén, 1820)

New records: Učka Nature Park, nr Vela Učka, 19.VI.2012, E. Buenaventura, T. Pape, D. Whitmore leg. (1♀) (NHMD).

Literature records: Orehovica (Langhoffer 1920); Baranov (1928); Zagreb (Sisojević et al. 1989).

European distribution: Andorra, Austria, Belarus, Belgium, Bulgaria, Croatia, Czech Republic, Denmark (mainland), Estonia, Finland, France (mainland), Germany, Greece (mainland), Hungary, Ireland, Italy (mainland, Sicily), Lithuania, Moldova, Norway (mainland), Poland, Romania, Russia (Central European Territory, North European Territory, South European Territory), Slovakia, Spain (mainland), Sweden, Switzerland, The Netherlands, Ukraine, United Kingdom.

##### 39. *Nyctiahalterata* (Panzer, 1798)

New records: Dubrovnik, Ombla, 27.V.1934, D. Aubertin leg. (1♂) (NHMUK); Brač Is., Bol, 4.VIII.2004, D. Whitmore leg. (1♂) (DW); Igrane, 19.V.2012, C. Lange, J. Ziegler leg. (1♂) (ZMHB); Učka Nature Park, nr Vela Učka, 19.VI.2012, E. Buenaventura, T. Pape, D. Whitmore leg. (12♂) (NHMD).

Literature records: Zagreb; Sljeme; Osijek; Bregi; Petrinja; Bakar; Orehovica; Riječina (Langhoffer 1920); Baranov (1928); Trnovec; Kaštel Stari; Komiža; Lopud; Makarska; Paklenica, Velebit Mts; Solin; Split; Trogir; Žrnovnica; Vozilići (Sisojević et al.1989).

European distribution: Albania, Austria, Belarus, Belgium, Bulgaria, Croatia, Cyprus, Czech Republic, Denmark (mainland), Estonia, France (mainland, Corsica), Germany, Greece (mainland), Hungary, Italy (mainland, Sicily), Latvia, Malta, Moldova, Poland, Romania, Russia (Central European Territory, South European Territory), Slovakia, Spain (mainland), The Netherlands, Ukraine, United Kingdom.

Remarks: We here record this species from Sicily for the first time (Palermo, Corleone, Rocca Busambra, 37°50'42.89"N, 13°23'10.37"E, 950 m, 30.VI.2005, P. Cerretti leg., 1♀, DW).

##### 40. *Nyctialugubris* (Macquart, 1843) (▲)

Records: Brač Is., Bol, 5.VIII.2004, D. Whitmore leg. (1♀) (DW).

European distribution: Croatia, Cyprus, France (mainland), Italy (mainland, Sardinia, Sicily); Malta, Portugal (mainland), Sicily, Spain (mainland).

Remarks: We here record this species from mainland Italy (Abruzzo; Calabria; Emilia-Romagna; Latium; 6♂, 3♀, DW and M. Mei collection) and Sardinia (Iglesias [outskirts of town], 23.V.2006, D. Whitmore leg., 1♂, DW; Iglesias, Mamenga, 610 m, 39°21'29.47"N, 8°33'39.24"E, 18.VII.2006, P. Cerretti, D. Whitmore leg., 3♀, 1♂, DW; Iglesias, Conca Margiani, 39°21'39.83"N, 8°33'50.46"E, 725 m, 7.IX.2006, D. Avesani et al. leg., 1♀, DW; San Vero Milis, Sa Marigosa, 40°2'24.99"N, 8°24'17.00"E, 13.IX.2006, D. Avesani et al. leg., 1♀, DW) for the first time.

##### 41. *Sarcophilalatifrons* (Fallén, 1817)

New records: Tribanj-Kruščica, Ljubotić, 14.VI.2012, E. Buenaventura, T. Pape, D. Whitmore leg. (2♀, 6♂) (NHMD).

Literature records: Rijeka; Hvar (Strobl 1893); Dubrovnik; Hvar; Krka Falls; Split (Strobl 1900); Zadar (Strobl 1904); Senj (Langhoffer 1920); Baranov (1928, 1938).

European distribution: Albania, Austria, Belarus, Belgium, Bulgaria, Croatia, Czech Republic, Denmark (mainland), France (mainland), Germany, Greece (mainland), Hungary, Italy (mainland), Lithuania, Luxemburg, Poland, Romania, Russia (Central European Territory, South European Territory), Slovakia, Spain (mainland, Canary Is.), Sweden, Switzerland, The Netherlands, Ukraine, United Kingdom.

##### 42. *Sarcophila* sp.

Records: Brač Is., Bol, 4.VIII.2004, D. Whitmore leg. (1♀) (DW); same locality, 5.VIII.2004, D. Whitmore leg. (4♀, 1♂) (DW); Hvar Is., Dubovica, 12.VIII.2004, D. Whitmore leg. (1♀, 1♂) (DW); Pakleni Is., Sveti Klement, 8.VIII.2004, D. Whitmore leg. (1♀) (DW).

Remarks: The above-listed specimens belong to a common and widespread Mediterranean species or species complex, of which we have examined numerous specimens also from Corsica, Greece (incl. Corfu), and southern and insular Italy. Pending the examination of type material in the framework of a full revision of the genus *Sarcophila* Rondani, we prefer to leave these specimens unnamed for the time being.

##### 43. *Wohlfahrtiamagnifica* (Schiner, 1862)

Literature records: Baranov (1928); Dubrovnik, nr Loznica; Osijek (Baranov 1943).

European distribution: Albania, Austria, Belarus, Bulgaria, Croatia, Cyprus, Czech Republic, France (mainland), Germany, Greece (mainland), Hungary, Italy (mainland, Sardinia, Sicily), Lithuania, Moldova, Poland, Portugal (mainland), Romania, Russia (Central European Territory, South European Territory), Serbia, Slovakia, Spain (mainland), Ukraine.

Remarks: Larvae recorded on sheep by Mikačić (1938) were identified as belonging to *W.magnifica*. However, these records should be treated with caution because the larvae of *Wohlfahrtia* Brauer & Bergenstamm are difficult to identify to species level.

##### 44. *Wohlfahrtiameigenii* (Schiner, 1862) (*)

Literature records: Langhoffer (1920); Baranov (1928).

European distribution: Austria, Belarus, Belgium, Bulgaria, Croatia, Czech Republic, Denmark (mainland), France (mainland), Germany, Hungary, Italy (mainland), Latvia, Lithuania, Moldova, Poland, Romania, Russia (Central European Territory, South European Territory), Slovakia, Spain (mainland), Switzerland, Ukraine.

Remarks: Unverified record. Although male and female terminalia provide important characters for distinguishing *W.meigenii* from other closely related species of *Wohlfahrtia*, misidentifications in older generalist publications cannot be ruled out. The taxonomy of the nominal taxa *W.meigenii* (Schiner) and *W.vigil* (Walker) is unsettled, as discussed by Hall et al. (2009) and Ge et al. (2018). We are here using *W.meigenii* due to its Palaearctic type locality and because this was the name used by Langhoffer (1920) and Baranov (1928).

#### Subfamily Sarcophaginae Macquart, 1834

##### 45. Blaesoxipha (Blaesoxipha) arenicola Rohdendorf, 1928

Literature records: Baranov (1931); Pag Is. (Baranov 1942); Pape (1996, 2004).

European distribution: Croatia, France (mainland), Switzerland.

##### 46. Blaesoxipha (Blaesoxipha) aurulenta Rohdendorf, 1937 (▲)

Records: Pag Is., 21.VI.1931, N. Baranov leg. (1♂) (NMNH); same locality, VII.1933 N. Baranov leg. (2♂) (NMNH); same locality, 19.VII.1934, N. Baranov leg. (1♂) (NMNH).

European distribution: Croatia, France (mainland).

##### 47. Blaesoxipha (Blaesoxipha) batilligera Séguy, 1941 (▲)

New records: Pag Is., 26.VII.1933, N. Baranov leg. (2♀, 1♂) (NMNH); Sljeme, 2.VIII.1929, N. Baranov leg. (2♂) (NMNH); same locality, 13.IX.1933, N. Baranov leg. (1♀) (NMNH).

Distribution: Croatia, France (mainland), Greece (mainland), Russia (Central European Territory), Switzerland.

##### 48. Blaesoxipha (Blaesoxipha) cochlearis (Pandellé, 1896)

Literature records: Otočac; Pag Is.; Sljeme (Baranov 1942).

European distribution: Bulgaria, Croatia, Czech Republic, France (mainland), Germany, Hungary, Italy (mainland), Poland, Russia (Central European Territory, South European Territory), Romania, Slovakia, Spain (mainland), Switzerland, Ukraine.

##### 49. Blaesoxipha (Blaesoxipha) lapidosa Pape, 1994

Literature records: Baranov (1938); Delnice; Krapina; Otočac; Požega; Sljeme; Zagreb (Baranov 1942, as *lineata* Fallén); Pape (1994, 1996, 2004).

European distribution: Albania, Austria, Bulgaria, Croatia, Czech Republic, Denmark (mainland), Finland, France (mainland, Corsica), Germany, Hungary, Italy (mainland, Sardinia, Sicily), Lithuania, Malta, Moldova, Norway (mainland), Poland, Romania, Russia (Central European Territory, South European Territory), Serbia, Slovakia, Slovenia, Spain (mainland, Canary Is.), Switzerland, The Netherlands, Ukraine.

Remarks: We here record this species from Sicily for the first time (Agrigento, 25.VI.1941, A. Giordani-Soika leg., 1♂, Museo di Zoologia, Sapienza Università di Roma, Rome).

##### 50. Blaesoxipha (Blaesoxipha) laticornis (Meigen, 1826)

Literature records: Koprivnica; Krapina; Požega; Samobor; Zagreb (Baranov 1942).

European distribution: Austria, Bulgaria, Croatia, Denmark (mainland), France (mainland), Germany, Italy (mainland), Liechtenstein, Poland, Serbia, Russia (Central European Territory, South European Territory), Switzerland, Ukraine.

##### 51. Blaesoxipha (Blaesoxipha) litoralis (Villeneuve, 1911)

Literature records: Pag Is. (Baranov 1942).

European distribution: Bulgaria, Croatia, France (mainland), Hungary, Italy (Sardinia), Russia (South European Territory), Serbia, Spain (mainland), Switzerland, Ukraine.

Remarks: We here record this species from Sardinia for the first time (Domusnovas, Valle Oridda, 4.IX.2003, P. Cerretti et al. leg., 2♂, DW; same as previous except 23.IX.2004, 1♂, DW; Villacidro, Canale Monincu, 39°25'10.01"N, 8°37'40.61"E, 450 m, 21.V.2006, D. Whitmore leg., 2♂, DW; Gonnosfanàdiga, Monte Idda, 39°28'11.72"N, 8°36'56.60"E, 474 m, 22.V.2006, D. Whitmore leg., 1♂, DW; Domusnovas, Valle Oridda, 39°24'32.16"N, 8°36'58.94"E, 592 m, 24.V.2006, D. Whitmore leg., 1♂, DW; same as previous except 15–17.VII.2006, 12♂, DW; Domusnovas, Rio Sa Duchessa, 39°21'41.85"N, 8°36'48.46"E, 270 m, 12.VII.2006, D. Whitmore leg., 1♂, DW), thus confirming it for Italy (see Pape et al. 1995).

##### 52. Blaesoxipha (Blaesoxipha) plumicornis (Zetterstedt, 1859) (▲)

Records: Krapina, 11.VII.1929, N. Baranov leg. (1♂) (NMNH); Samobor, 22.IX.1930, N. Baranov leg. (1♂) (NMNH); Zagreb, 30.VIII.1929, N. Baranov leg. (1♂) (NMNH); same locality, 1.IX.1929, N. Baranov leg. (1♂) (NMNH); same locality, 25.VII.1931, N. Baranov leg. (1♂) (NMNH).

European distribution: Albania, Austria, Belarus, Belgium, Bulgaria, Croatia, Czech Republic, Denmark (mainland), Estonia, Finland, France (mainland, Corsica), Germany, Hungary, Italy (mainland, Sicily), Latvia, Norway (mainland), Poland, Russia (Central European Territory, South European Territory), Serbia, Slovakia, Sweden, Switzerland, The Netherlands, Ukraine, United Kingdom.

Remarks: We here record this species from Sicily for the first time (Palermo, Parco delle Madonie, 29.VI.2005, D. Whitmore leg., 2♂, DW; Palermo, Corleone, Rocca Busambra, 37°50'42.89"N, 13°23'10.37"E, 950 m, 30.VI.2005, D. Whitmore leg., 1♂, DW).

##### 53. Blaesoxipha (Blaesoxipha) pygmaea (Zetterstedt, 1844)

Literature records: Pape (1994, 1996, 2004).

European distribution: Croatia, Denmark (mainland), France (mainland), Germany, Italy (mainland), Poland, Switzerland.

##### 54. Blaesoxipha (Blaesoxipha) ungulata (Pandellé, 1896)

Literature records: Krapina; Zagreb (Baranov 1942).

European distribution: Andorra, Bulgaria, Croatia, Czech Republic, France (mainland), Hungary, Italy (mainland, Sardinia, Sicily), Poland, Serbia, Russia (Central European Territory, South European Territory), Spain (mainland), Switzerland, Ukraine.

Remarks: We here record this species from Sardinia (Domusnovas, Bega d’Aleni, 39°24'1.76"N, 8°37'33.66"E, 621 m, 17.VII.2006, D. Whitmore leg., 1♀, 1♂, DW) and Sicily (Palermo, Bosco della Ficuzza, 24.VI.2005, D. Whitmore leg., 1♀, DW) for the first time.

##### 55. Blaesoxipha (Blaesoxipha) unicolor (Villeneuve, 1912)

Literature records: Zagreb (Baranov 1942, as *intermedia* Baranov); Pape (1996, 2004).

European distribution: Andorra, Bulgaria, Croatia, France (mainland), Hungary, Italy (mainland, Sardinia), Russia (Central European Territory, South European Territory), Serbia, Spain (mainland), Switzerland, Ukraine.

Remarks: We here record this species from Sardinia for the first time (Domusnovas, nr Agriturismo Perda Niedda, 350 m, 8.VI.2004, P. Cerretti et al. leg., 1♂, DW; Domusnovas, Sa Duchessa, 39°22'27.18"N, 8°35'36.74"E, 371 m, 12.VI.2006, D. Whitmore leg., 1♂, DW).

##### 56. Blaesoxipha (Servaisia) croatica Baranov, 1942

Literature records: Zagreb (Baranov 1942, as *silantjevi* ssp. croatica); Pape (1996, 2004).

Distribution: Croatia.

##### 57. Blaesoxipha (Servaisia) erythrura (Meigen, 1826)

New records: Podoštra, 15.VI.2012, E. Buenaventura, T. Pape, D. Whitmore leg. (1♂) (NHMD); Baške Oštarije, 13.VI.2012, E. Buenaventura, T. Pape, D. Whitmore leg. (2♂, 1♀) (NHMD); Sušanj Cesarički, 13.VI.2012, E. Buenaventura, T. Pape, D. Whitmore leg. (1♂) (NHMD).

Literature records: Zadar (Strobl 1904); Mraclin; Samobor; Zagreb (Baranov 1942).

European distribution: Austria, Belarus, Belgium, Bulgaria, Croatia, Czech Republic, Estonia, Finland, France (mainland), Germany, Greece (mainland), Hungary, Italy (mainland), Lithuania, Poland, Russia (Central European Territory, South European Territory), Serbia, Slovakia, Spain (mainland), Sweden, Switzerland, Ukraine, United Kingdom.

##### 58. Blaesoxipha (Servaisia) rossica Villeneuve, 1912

New records: Vrhovine, 3.VII.1955, R.L. Coe leg. (2♂) (NHMUK); Zmajevac, 15.VII.2015, S. Krčmar leg. (1♂) (DBUO); same locality, 25.VIII.2017, S. Krčmar leg. (2♂) (DBUO).

Literature records: Delnice; Mraclin; Mrzla Vodica; Požega; Samobor; Zagreb (Baranov 1942).

European distribution: Albania, Austria, Bulgaria, Croatia, Czech Republic, Denmark (mainland), Estonia, France (mainland), Germany, Hungary, Italy (mainland), Norway (mainland), Poland, Romania, Russia (Central European Territory, South European Territory), Serbia, Slovakia, Switzerland, Ukraine, United Kingdom.

##### 59. Blaesoxipha (Tephromyia) grisea (Meigen, 1826)

New records: Pag Is., VIII.1934, N. Baranov leg. (1♂) (NMNH); same locality, VII.1935, N. Baranov leg. (4♀) (NMNH); Samobor, 19.VI.1931, N. Baranov leg. (2♂) (NMNH); Sljeme, 23.VI.1931, N. Baranov leg. (2♂) (NMNH).

Literature records: Baranov (1931); Krapina; Zagreb (Baranov 1942).

European distribution: Albania, Austria, Belarus, Belgium, Bulgaria, Croatia, Czech Republic, France (mainland), Germany, Hungary, Italy (mainland), Moldova, Poland, Romania, Russia (Central European Territory, South European Territory), Serbia, Slovakia, Slovenia, Spain (mainland), Switzerland, Ukraine.

##### 60. *Raviniapernix* (Harris, 1780)

New records: Dalmatia, 18–19.V.1927, T. Becker leg. (2♂) (ZMHB); Korčula, 22–27.V.1955, R.L. Coe leg. (1♂, 4♀) (NHMUK).

Literature records: Dubrovnik; Hvar; Krka Falls; Solin; Šibenik (Strobl 1900, as *haematodes* Meigen); Zadar (Strobl 1904, as *haematodes*); Bakar; Klana; Samobor; Senj; Zagreb (Langhoffer 1920, as *haematodes*); Baranov (1928, as *haematodes*); Krapina (Baranov 1929, as *striata* Fabricius); Baranov (1938, as *striata*); Pag Is., Metajna (Baranov 1940, 1941a, as *striata*); Gruž; Krapina; Pag Is.; Zagreb (Baranov 1942, as *striata*); Trogir (Strukan 1964, as *striata*); Gotalovo (Sisojević et al. 1989); Podgora (Povolný and Znojil 1998, 1999).

European distribution: Albania, Andorra, Austria, Belarus, Belgium, Bulgaria, Croatia, Cyprus, Czech Republic, Denmark (mainland), Estonia, Finland, France (mainland, Corsica), Germany, Greece (mainland), Hungary, Italy (mainland, Sardinia, Sicily), Latvia, Lithuania, Malta, Moldova, Norway (mainland), Poland, Portugal (mainland), Romania, Russia (Central European Territory, South European Territory), Serbia, Slovakia, Spain (mainland, Canary Is.), Sweden, Switzerland, The Netherlands, Ukraine, United Kingdom.

##### 61. Sarcophaga (Bellieriomima) subulata Pandellé, 1896

New records: Baške Oštarije, 13.VI.2012, E. Buenaventura, T. Pape, D. Whitmore leg. (1♂) (NHMD); Podoštra, 15.VI.2012, E. Buenaventura, T. Pape, D. Whitmore leg. (1♂) (NHMD); Čigoč, 10.VI.2016, S. Krčmar leg. (1♂) (DBUO); same locality, 5.VI.2017, S. Krčmar leg. (3♂) (DBUO); Skrad, 11.VI.2016, S. Krčmar leg. (3♂) (DBUO); same locality, 31.V.2017, S. Krčmar leg. (6♂) (DBUO); same locality, 8.VI.2017, S. Krčmar leg. (14♂) (DBUO); Zmajevac, 15.VII.2015, S. Krčmar leg. (1♂) (DBUO); same locality, 9.VIII.2016, S. Krčmar leg. (1♂) (DBUO); same locality, 30.IV.2017, S. Krčmar leg. (2♂) (DBUO); same locality, 14.V.2017, S. Krčmar leg. (3♂) (DBUO); same locality, 11.IX.2017, S. Krčmar leg. (4♂) (DBUO).

Literature records: Zagreb; Mraclin; Samobor (Baranov 1942, as *laciniata* Pandellé); Otočac, Veliki Kuk (Rucner 1994); Pape (2004).

European distribution: Austria, Belarus, Belgium, Bulgaria, Croatia, Czech Republic, Denmark (mainland), Estonia, Finland, France (mainland), Germany, Hungary, Italy (mainland), Norway (mainland), Poland, Romania, Russia (Central European Territory, North European Territory, South European Territory), Slovakia, Spain (mainland), Sweden, Switzerland, The Netherlands, Ukraine, United Kingdom.

##### 62. Sarcophaga (Bercaea) africa (Wiedemann, 1824)

New records: Korčula, 22–27.V.1955, R.L. Coe leg. (5♂, 10♀) (NHMUK); Hvar Is., Dubovica, 12.VIII.2004, D. Whitmore leg. (1♀) (DW); Sveti Juraj, 12.VI.2012, E. Buenaventura, T. Pape, D. Whitmore leg. (1♂) (NHMD); Drivenik, 5.VI.2014, S. Krčmar leg. (1♂) (DBUO); Komin, 16.VII.2014, S. Krčmar leg. (3♂) (DBUO); Badžula, 18.VII.2014, S. Krčmar leg. (1♂) (DBUO); Biograd, 18.VIII.2014, S. Krčmar leg. (1♂) (DBUO); same locality, 19.VIII.2014, S. Krčmar leg. (4♂) (DBUO); same locality, 20.VIII.2014, S. Krčmar leg. (2♂) (DBUO); Krk Is., Punat, 13.VI.2016, S. Krčmar leg. (1♂) (DBUO); Zmajevac, 11.IX.2017, S. Krčmar leg. (1♂) (DBUO).

Literature records: Dubrovnik; Šibenik; Zadar (Strobl 1900, as *nurus* Rondani); Solin; Split (Strobl 1900, as *nurus*); Dalmatia (Böttcher 1913, as *haemorrhoidalis* Meigen); Baranov (1928, as *haemorrhoidalis*); Pag Is., Metajna (Baranov 1940, as *haemorrhoidalis*); Gruž; Korčula; Pag Is.; Zagreb; Samobor; Krapina (Baranov 1942, as *haemorrhoidalis*); Donja Stubica; Trogir; Velika Paklenica (Strukan 1970, as *haemorrhoidalis*); Oprić (Sisojević et al. 1989, as *cruentata* Meigen); Pelješac, Potomje; Ston, Česvinica; Ston, Broce (Rucner 1994, as *haemorrhoidalis*); Biokovo Mts; Podgora (Povolný and Znojil 1994, 1998, 1999, as *cruentata*); Krk Is. (Povolný and Znojil 1998, as *cruentata*); Pape (2004).

European distribution: Albania, Austria, Belarus, Belgium, Bulgaria, Croatia, Cyprus, Czech Republic, Denmark (mainland), France (mainland, Corsica), Germany, Greece (mainland), Hungary, Ireland, Italy (mainland, Sardinia, Sicily), Latvia, Lithuania, Luxemburg, Moldova, Norway (mainland), Poland, Portugal (mainland, Madeira Is.), Romania, Russia (Central European Territory, South European Territory), Serbia, Slovakia, Spain (mainland, Canary Is.), Sweden, Switzerland, The Netherlands, Ukraine, United Kingdom.

##### 63. Sarcophaga (Helicophagella) agnata Rondani, 1860

New records: Baške Oštarije, 13.VI.2012, E. Buenaventura, T. Pape, D. Whitmore leg. (2♂) (NHMD); Sušanj Cesarički, 13.VI.2012, E. Buenaventura, T. Pape, D. Whitmore leg. (1♂) (NHMD); Plitvička jezera National Park, Turčić, 16.VI.2012, E. Buenaventura, T. Pape, D. Whitmore leg. (1♂) (NHMD).

Literature records: Zagreb; Našice (Baranov 1942); Pape (2004).

Distribution: Albania, Austria, Belgium, Bulgaria, Croatia, Czech Republic, Denmark (mainland), France (mainland, Corsica), Germany, Hungary, Italy (mainland, Sardinia, Sicily), Norway (mainland), Poland, Romania, Russia (Central European Territory), Slovakia, Spain (mainland), Sweden, Switzerland, The Netherlands, Ukraine, United Kingdom.

Remarks: We here record this species from Sardinia for the first time (Domusnovas, Conca Margiani, 39°21'37.59"N, 8°33'58.62"E, 700 m, 16–17.VII.2006, D. Whitmore leg., 5♂, DW; same as previous except 39°21'40.76"N, 8°33'44.05"E, 750 m, 2♂, DW).

##### 64. Sarcophaga (Helicophagella) crassimargo Pandellé, 1896

New records: Zmajevac, 11.IX.2017, S. Krčmar leg. (2♂) (DBUO).

Literature records: Zagreb; Krapina; Požega (Baranov 1942); Pape (2004).

European distribution: Albania, Austria, Belgium, Bulgaria, Croatia, Czech Republic, Denmark (mainland), Finland, France (mainland), Germany, Greece (mainland), Hungary, Ireland, Italy (mainland), Lithuania, Macedonia, Moldova, Norway (mainland), Poland, Romania, Russia (Central European Territory, South European Territory), Serbia, Slovakia, Spain (mainland), Sweden, Switzerland, The Netherlands, Ukraine, United Kingdom.

##### 65. Sarcophaga (Helicophagella) hirticrus Pandellé, 1896

New records: Brač Is., Bol, 4.VIII.2004, D. Whitmore leg. (1♂) (DW); Sveti Juraj, 12.VI.2012, E. Buenaventura, T. Pape, D. Whitmore leg. (1♂) (NHMD); Zmajevac, 2.VII.2014, S. Krčmar leg. (1♂) (DBUO); same locality, 4.VII.2014, S. Krčmar leg. (1♂) (DBUO).

Literature records: Baranov (1928); Gruž; Otočac; Pag Is.; Zagreb (Baranov 1942); Biokovo Mts; Podgora (Povolný and Znojil 1998, 1999); Pape (2004).

European distribution: Albania, Andorra, Austria, Belgium, Bulgaria, Croatia, Czech Republic, France (mainland, Corsica), Germany, Hungary, Italy (mainland, Sardinia, Sicily), Malta, Norway (mainland), Poland, Romania, Russia (South European Territory), Slovakia, Spain (mainland, Canary Is.), Sweden, Switzerland, Ukraine, United Kingdom.

Remarks: This species was not listed for Sardinia by Pape (2004), but has been known from the island at least since Povolný (1997).

##### 66. Sarcophaga (Helicophagella) melanura Meigen, 1826

New records: Novi Varoš, 27.VII.1969, W.G. Tremewan leg. (1♀) (NHMUK); Modro Oko, 18.VII.2014, S. Krčmar leg. (1♂) (DBUO); Zmajevac, 14.V.2017, S. Krčmar leg. (1♂) (DBUO); same locality, 10.VIII.2017, S. Krčmar leg. (2♂) (DBUO); same locality, 11.IX.2017, S. Krčmar leg. (3♂) (DBUO).

Literature records: Hvar; Split (Strobl 1900); Zadar (Strobl 1904); Bakar; Bjelovar; Delnice; Osijek; Pleskovac; Prezid; Senj; Zagreb (Langhoffer 1920); Baranov (1928); Krapina; Pag Is.; Zagreb; Mraclin; Samobor (Baranov 1942); Oprić (Sisojević et al. 1989); Podgora (Povolný and Znojil 1994, 1998, 1999); Pape (2004).

European distribution: Albania, Austria, Belarus, Belgium, Bulgaria, Croatia, Cyprus, Czech Republic, Denmark (mainland), Finland, France (mainland, Corsica), Germany, Greece (mainland), Hungary, Ireland, Italy (mainland, Sardinia, Sicily), Latvia, Lithuania, Luxemburg, Macedonia, Malta, Moldova, Norway (mainland), Poland, Portugal (mainland), Romania, Russia (Central European Territory, South European Territory), Serbia, Slovakia, Spain (mainland, Canary Is.), Sweden, Switzerland, The Netherlands, Ukraine, United Kingdom.

##### 67. Sarcophaga (Helicophagella) novella Baranov, 1929

Literature records: Biokovo Mts (Povolný and Verves 1997).

Distribution: Croatia, France (Corsica), Hungary, Italy (mainland, Sardinia), Macedonia, Romania, Serbia.

Remarks: A junior synonym of this species, *Helicophagellareicostae* Povolný, 1999, was described from Sardinia. It has since been confirmed as locally abundant on the island (Whitmore, unpubl. data).

##### 68. Sarcophaga (Helicophagella) noverca Rondani, 1860

New records: Podoštra, 15.VI.2012, E. Buenaventura, T. Pape, D. Whitmore leg. (3♂) (NHMD); Sušanj Cesarički, 13.VI.2012, E. Buenaventura, T. Pape, D. Whitmore leg. (1♂) (NHMD); Kamenac, 23.VII.2016, S. Krčmar leg. (1♂) (DBUO); Zmajevac, 27.VI.2014, S. Krčmar leg. (2♂) (DBUO); same locality, 2.VII.2014, S. Krčmar leg. (1♂) (DBUO); same locality, 4.VII.2014, S. Krčmar leg. (4♂) (DBUO); same locality, 30.IV.2017, S. Krčmar leg. (2♂) (DBUO); Skrad, 8.VI.2017, S. Krčmar leg. (1♂) (DBUO).

Literature records: Dubrovnik; Šibenik (Strobl 1900); Zadar (Strobl 1904); Gruž; Krapina; Mraclin; Sljeme; Zagreb (Baranov 1942); Medvednica, Sv. Jakob; Medevednica, Medvedgrad; Otočac, Metla; Otočac, Veliki Kuk; Baške Oštarije, Filipov Kuk (Rucner 1994); Podgora (Povolný and Znojil 1998, 1999); Pape (2004).

European distribution: Albania, Austria, Belgium, Bulgaria, Croatia, Czech Republic, France (mainland, Corsica), Germany, Hungary, Italy (mainland), Macedonia, Malta, Norway (mainland), Poland, Romania, Russia (South European Territory), Serbia, Slovakia, Spain (mainland), Sweden, Switzerland, The Netherlands, Ukraine.

##### 69. Sarcophaga (Helicophagella) novercoides Böttcher, 1913

New records: Sveti Juraj, 12.VI.2012, E. Buenaventura, T. Pape, D. Whitmore leg. (1♂) (NHMD).

Literature records: Biokovo Mts (Povolný and Znojil 1998, 1999); Podgora (Povolný and Znojil 1994, 1998, 1999); Pape (2004).

European distribution: Albania, Austria, Bulgaria, Croatia, Cyprus, France (mainland), Germany, Greece (mainland), Hungary, Italy (mainland, Sardinia, Sicily), Malta, Russia (South European Territory), Serbia, Slovakia, Spain (mainland), Switzerland, Ukraine.

##### 70. Sarcophaga (Helicophagella) okaliana (Lehrer, 1975) (▲)

Records: Begovo Razdolje, nr Bijele Stijene, 18.VI.2012, E. Buenaventura, T. Pape, D. Whitmore leg. (1♂) (NHMD); Bjelolasica Mts, 2.VI.2015, S. Krčmar leg. (1♂) (DBUO).

Distribution: Albania, Austria, Croatia, France (mainland), Italy (mainland), Slovakia, Spain (mainland), Switzerland.

##### 71. Sarcophaga (Helicophagella) rosellei Böttcher, 1912

New records: Podoštra, 15.VI.2012, E. Buenaventura, T. Pape, D. Whitmore leg. (2♂) (NHMD); Plitvička jezera National Park, Turčić, 16.VI.2012, E. Buenaventura, T. Pape, D. Whitmore leg. (2♂) (NHMD); Begovo Razdolje, nr Bijele Stijene, 18.VI.2012, E. Buenaventura, T. Pape, D. Whitmore leg. (1♂) (NHMD); Oštrovica, 13.VI.2016, S. Krčmar leg. (1♂) (DBUO); Skrad, 31.V.2017, S. Krčmar leg. (1♂) (DBUO); same locality, 8.VI.2017, S. Krčmar leg. (1♂) (DBUO); Lokve, 8.VI.2017, S. Krčmar leg. (2♂) (DBUO).

Literature records: Delnice; Mrzla Vodica; Samobor; Sljeme; Zagreb (Baranov 1942); Pape (2004).

European distribution: Austria, Belgium, Bulgaria, Croatia, Czech Republic, Denmark (mainland), France (mainland), Germany, Hungary, Italy (mainland, Sicily), Norway (mainland), Poland, Romania, Slovakia, Spain (mainland), Sweden, Switzerland, The Netherlands, Ukraine, United Kingdom.

##### 72. Sarcophaga (Heteronychia) amita Rondani, 1860 (▲)

Records: Krk Is., Punat, 7.VI.2017, S. Krčmar leg. (1♂) (DBUO).

Literature records: Dubrovnik; Krka Falls; Šibenik (Strobl 1900, as *haemorrhoa* var. amita). NB: These records could not be assigned to this species with certainty.

Distribution: Croatia, France (mainland, Corsica), Germany, Hungary, Italy (mainland), Spain (mainland), Switzerland.

##### 73. Sarcophaga (Heteronychia) ancilla Rondani, 1865 (▲)

Records: Sušanj Cesarički, 13.VI.2012, E. Buenaventura, T. Pape, D. Whitmore leg. (1♂) (NHMD).

European distribution: Austria, Bulgaria, Croatia, Czech Republic, France (mainland), Greece (mainland), Hungary, Italy (mainland), Romania, Russia (Central European Territory, South European Territory), Serbia, Slovakia, Spain (mainland), Switzerland, Ukraine. NB: Some of these country-level records require verification (see Whitmore 2010).

##### 74. Sarcophaga (Heteronychia) arcipes Pandellé, 1896

New records: Podoštra, 15.VI.2012, E. Buenaventura, T. Pape, D. Whitmore leg. (1♂) (NHMD).

Literature records: Krapina; Otočac (Baranov 1942); Bosut (Strukan 1967); Pape (2004).

Distribution: Austria, Belgium, Bulgaria, Croatia, Czech Republic, France (mainland), Germany, Hungary, Italy (mainland), Poland, Romania, Serbia, Slovakia, Spain (mainland), Switzerland, The Netherlands, Ukraine, United Kingdom.

##### 75. Sarcophaga (Heteronychia) belanovskyi (Verves, 1973)

Literature records: Krapina (Baranov 1942, as *ancilla* Rondani); Whitmore (2010).

European distribution: Croatia, Hungary, Italy (mainland), Romania, Serbia, Ukraine.

##### 76. Sarcophaga (Heteronychia) benaci Böttcher, 1913

New records: Sušanj Cesarički, 13.VI.2012, E. Buenaventura, T. Pape, D. Whitmore leg. (1♂) (NHMD); Učka Nature Park, Vela Učka, 19.VI.2012, E. Buenaventura, T. Pape, D. Whitmore leg. (1♂) (NHMD); Banja, 30.VI.2015, S. Krčmar leg. (1♂) (DBUO); Desne, 1.VII.2015, S. Krčmar leg. (1♂) (DBUO); Vriještica, 3.VII.2015, S. Krčmar leg. (1♂) (DBUO); Zmajevac, 15.VII.2017, S. Krčmar leg. (1♂) (DBUO).

Literature records: Pag Is. (Baranov 1942); Biokovo Mts, Čerešnik nr Makarska (Povolný 1987, as *vachai* Povolný); Biokovo Mts; Podgora (Povolný and Znojil 1994, as *bezziana* Böttcher, misidentification); Pape (1996, as *vachai*); Povolný and Verves (1997, as *bezziana*); Biokovo Mts; Podgora (Povolný and Znojil 1998, 1999); Pape (2004, as *vachai*); Biokovo Mts, nr Podgora; Hvar Is., Jelsa; Krk Is., Veli Vrh (Whitmore 2011).

Distribution: Bulgaria, Croatia, Czech Republic, Germany, Greece (mainland), Italy (mainland), Serbia, Slovakia, Spain (mainland).

##### 77. Sarcophaga (Heteronychia) boettcheri Villeneuve, 1912

Literature records: Zagreb, Stenjevac; Pag Is. (Baranov 1942); Pape (2004); Pag Is.; Stinica (Whitmore 2011).

Distribution: Austria, Croatia, Cyprus, Greece (mainland, Cyclades Is.), Hungary, Romania, Serbia, Ukraine.

##### 78. Sarcophaga (Heteronychia) bulgarica (Enderlein, 1936)

New records: Baške Oštarije, 13.VI.2012, E. Buenaventura, T. Pape, D. Whitmore leg. (1♂) (NHMD); Plitvička jezera National Park, Turčić, 16.VI.2012, E. Buenaventura, T. Pape, D. Whitmore leg. (1♂) (NHMD).

Literature records: Pape (2004).

European distribution: Austria, Belarus, Bulgaria, Croatia, Czech Republic, Denmark (mainland), Estonia, France (mainland, Corsica), Germany, Hungary, Italy (mainland, Sardinia, Sicily), Moldova, Norway (mainland), Poland, Romania, Russia (Central European Territory, South European Territory), Slovakia, Spain (mainland), Sweden, Switzerland, Ukraine.

##### 79. Sarcophaga (Heteronychia) consanguinea Rondani, 1860

Literature records: Zadar (Strobl 1904); Zagreb (Baranov 1942, as *rondanii* Böttcher); Verves and Khrokalo (2014).

European distribution: Bulgaria, Croatia, France (mainland), Greece (mainland), Hungary (doubtful), Italy (mainland, Sicily), Russia (South European Territory), Serbia, Ukraine.

##### 80. Sarcophaga (Heteronychia) croca Pape, 1996

New records: Sušanj Cesarički, 13.VI.2012, E. Buenaventura, T. Pape, D. Whitmore leg. (1♂) (NHMD).

Literature records: Biokovo Mts, nr Podgora; Hvar Is., nr Jelsa (Whitmore 2011).

Distribution: Croatia, Greece (mainland).

##### 81. Sarcophaga (Heteronychia) cucullans Pandellé, 1896

New records: Podoštra, 15.VI.2012, E. Buenaventura, T. Pape, D. Whitmore leg. (1♂) (NHMD).

Literature records: Otočac; Pag Is.; Zagreb (Baranov 1942); Metković, Šibanica (Rucner 1994); Pape (2004); Krk Is., Veli Vrh; Zadar, Borik; Zagreb (Whitmore et al. 2013).

European distribution: Austria, Bosnia and Herzegovina, Bulgaria, Croatia, Czech Republic, France (mainland), Germany, Greece (mainland), Hungary, Italy (mainland, Sicily), Russia (South European Territory), Serbia, Slovakia, Spain (mainland), Switzerland, Ukraine.

##### 82. Sarcophaga (Heteronychia) depressifrons Zetterstedt, 1845

New records: Sveti Juraj, 12.VI.2012, E. Buenaventura, T. Pape, D. Whitmore leg. (1♂) (NHMD); Skrad, 8.VI.2017, S. Krčmar leg. (1♂) (DBUO).

Literature records: Dubrovnik; Solin; Split (Strobl 1900); Samobor; Zagreb (Baranov 1942, as *offuscata* Meigen); Pape (2004); Krk Is., Glavotok; Labin; Sljeme; Zagreb (Whitmore 2011).

European distribution: Albania, Austria, Belarus, Belgium, Bosnia and Herzegovina, Bulgaria, Croatia, Czech Republic, Denmark (mainland), Estonia, Finland, France (mainland), Germany, Greece (mainland), Hungary, Italy (mainland), Macedonia, Malta, Norway (mainland), Poland, Romania, Russia (Central European Territory, South European Territory), Serbia, Slovakia, Spain (mainland), Sweden, Switzerland, The Netherlands, Ukraine, United Kingdom.

##### 83. Sarcophaga (Heteronychia) dissimilis Meigen, 1826

Literature records: Krapina (Baranov 1942); Virovitica (Sisojević et al. 1989); Pape (2004).

European distribution: Austria, Belarus, Belgium, Bulgaria, Croatia, Czech Republic, Estonia, France (mainland), Germany, Hungary, Italy (mainland), Latvia, Macedonia, Moldova, Poland, Romania, Russia (Central European Territory, South European Territory), Serbia, Slovakia, Spain (mainland), Switzerland, The Netherlands, Ukraine, United Kingdom.

##### 84. Sarcophaga (Heteronychia) filia Rondani, 1860

New records: Novi Grad, 27–31.V.1958, R.L. Coe leg. (1♀) (NHMUK); Brušane, 15.VI.2012, E. Buenaventura, T. Pape, D. Whitmore leg. (1♂) (NHMD); Krk Is., Punat, 1.VI.2017, S. Krčmar leg. (7♂) (DBUO); same locality, 7.VI.2017, S. Krčmar leg. (3♂) (DBUO).

Literature records: Baranov (1928); Krapina; Mraclin; Pag Is.; Susedgrad (Baranov 1938); Krapina; Pag Is.; Zagreb (Baranov 1942); Premuda; Bačinci (Strukan 1964); Oprić (Sisojević et al. 1989); Podgora (Povolný and Znojil 1994, 1998, 1999); Biokovo Mts; Pag Is.; Podgora; Zagreb (Whitmore 2011).

European distribution: Albania, Austria, Belgium, Bulgaria, Croatia, Czech Republic, France (mainland, Corsica), Germany, Greece (mainland, Crete), Hungary, Italy (mainland, Sardinia), Macedonia, Malta, Moldova, Poland, Romania, Russia (Central European Territory, South European Territory), Serbia, Slovakia, Spain (mainland), Switzerland, The Netherlands, Ukraine, United Kingdom.

##### 85. Sarcophaga (Heteronychia) giganta Pape, 1996

New records: Sveti Juraj, 12.VI.2012, E. Buenaventura, T. Pape, D. Whitmore leg. (1♂) (NHMD); same locality, 13.VI.2012, E. Buenaventura, T. Pape, D. Whitmore leg. (1♂) (NHMD).

Literature records: Biokovo Mts, Čerešnik nr Makarska (Povolný 1987, as *gigas* Povolný); Biokovo Mts; Podgora (Povolný and Znojil 1994, 1998, 1999, as *gigas*); Pape (1996); Dalmatia (Povolný 1996, as *gigas*); Pape (2004, as *gigas*); Biokovo Mts, nr Podgora (Whitmore 2011).

Distribution: Croatia.

##### 86. Sarcophaga (Heteronychia) haemorrhoa Meigen, 1826

New records: Podoštra, 15.VI.2012, E. Buenaventura, T. Pape, D. Whitmore leg. (2♂) (NHMD); Zmajevac, 15.VII.2015, S. Krčmar leg. (1♂) (DBUO); same locality, 30.IV.2017, S. Krčmar leg. (1♂) (DBUO).

Literature records: Dalmatia (Böttcher 1913); Zagreb (Langhoffer 1920); Dubrovnik; Krapina; Mraclin; Pag Is.; Zagreb, Podsused; Samobor (Baranov 1942); Podgora (Povolný and Znojil 1998, 1999); Krapina; Zagreb (Whitmore 2011).

European distribution: Albania, Austria, Belarus, Belgium, Bulgaria, Croatia, Czech Republic, Denmark (mainland), Estonia, Finland, France (mainland, Corsica), Germany, Greece (mainland), Hungary, Ireland, Italy (mainland, Sicily), Latvia, Norway (mainland), Poland, Romania, Russia (Central European Territory), Serbia, Slovakia, Spain (mainland), Sweden, Switzerland, The Netherlands, Ukraine, United Kingdom.

##### 87. Sarcophaga (Heteronychia) haemorrhoides Böttcher, 1913

New records: Novi Grad, 27–31.V.1958, R.L. Coe leg. (2♂, 1♀) (NHMUK); Baške Oštarije, 13.VI.2012, E. Buenaventura, T. Pape, D. Whitmore leg. (1♂) (NHMD); Tribanj-Krušćica, 14.VI.2012, E. Buenaventura, T. Pape, D. Whitmore leg. (1♂) (NHMD); Biograd, 20.VIII.2014, S. Krčmar leg. (1♂) (DBUO); Banja, 30.VI.2015, S. Krčmar leg. (1♂) (DBUO).

Literature records: Crikvenica; Fužine; Osijek; Senj; Zagreb (Langhoffer 1920); Bačinci (Strukan 1964); Podgora (Povolný and Znojil 1998, 1999); Pape (2004); Gračac; Krapina; Krk Is., Glavotok; Pakleni Is., Sveti Klement; Pag Is. (Whitmore et al. 2013).

European distribution: Albania, Austria, Belarus, Belgium, Bulgaria, Croatia, Czech Republic, Estonia, France (mainland), Germany, Greece (mainland), Hungary, Italy (mainland), Macedonia, Malta, Moldova, Poland, Romania, Russia (Central European Territory, South European Territory), Serbia, Slovakia, Spain (mainland), Switzerland, Ukraine.

##### 88. Sarcophaga (Heteronychia) infantilis Böttcher, 1913

New records: Sušanj Cesarički, 13.VI.2012, E. Buenaventura, T. Pape, D. Whitmore leg. (1♂) (NHMD).

Literature records: Sljeme (Whitmore et al. 2013).

European distribution: Austria, Bulgaria, Croatia, Czech Republic, France (mainland), Greece (mainland), Germany, Italy (mainland), Norway (mainland), Poland, Romania, Serbia, Slovakia, Spain (mainland), Sweden, Switzerland.

##### 89. Sarcophaga (Heteronychia) mediterranea Whitmore, 2011

Literature records: Pag Is. (Baranov 1942, as *penicillata* Villeneuve); Brač Is., Bol; Pag Is. (Whitmore 2011).

Distribution: Croatia, Italy (mainland, Sicily).

##### 90. Sarcophaga (Heteronychia) minima Rondani, 1862

New records: Rudelić Draga, 14.VI.2012, E. Buenaventura, T. Pape, D. Whitmore leg. (1♂) (NHMD).

Literature records: Pape (2004).

European distribution: Bulgaria, Croatia, Czech Republic, France (mainland, Corsica), Greece (mainland), Hungary, Italy (mainland, Sardinia, Sicily), Malta, Slovakia, Spain (mainland).

##### 91. Sarcophaga (Heteronychia) mutila Villeneuve, 1912

New records: Korčula, 22–27.V.1955, R.L. Coe leg. (10♂, 2♀) (NHMUK).

Literature records: Dalmatia (Böttcher 1913, as *setinervis* var. mutila); Gruž; Pag Is. (Baranov 1942, as “*mutilla*”); Biokovo Mts (Povolný and Znojil 1998, 1999); Premuda (Strukan 1964); Pape (2004); Krk Is., Glavotok; Pag Is. (Whitmore 2011).

European distribution: Bulgaria, Croatia, Cyprus, Greece (mainland), Hungary, Italy (mainland), Romania, Russia (South European Territory), Serbia, Slovakia, Ukraine.

##### 92. Sarcophaga (Heteronychia) pandellei (Rohdendorf, 1937)

Literature records: Dalmatia (Baranov 1942, as *consanguinea* Pandellé); Pape (2004); Brač Is., Bol; Hvar Is., Jelsa (Whitmore et al. 2013).

European distribution: Andorra, Croatia, France (mainland, Corsica), Greece (mainland), Italy (mainland, Sardinia, Sicily), Portugal (mainland), Spain (mainland).

Remarks: We here record this species from Greece for the first time (Corfu, Corfu city, 39°37'24.82"N, 19°55'19.76"E, 9.IX.2016, D. Whitmore leg., 1♂, DW).

##### 93. Sarcophaga (Heteronychia) pauciseta Pandellé, 1896

Literature records: Povolný and Verves (1997)

European distribution: Bulgaria, Croatia, Estonia, Germany, Hungary (doubtful), Poland, Russia (Central European Territory), Slovakia, Switzerland, Ukraine.

##### 94. Sarcophaga (Heteronychia) porrecta Böttcher, 1913

New records: Begovo Razdolje, nr Bijele Stijene, 18.VI.2012, E. Buenaventura, T. Pape, D. Whitmore leg. (1♂) (NHMD).

Literature records: Krapina; Medvedgrad; Samobor; Zagreb (Baranov 1942); Pape (2004); Krapina; Zagreb (Whitmore 2011); Zagreb, Samobor (Whitmore et al. 2013).

Distribution: Bulgaria, Croatia, Greece (mainland), Italy (mainland), Romania, Serbia, Slovakia.

##### 95. Sarcophaga (Heteronychia) proxima Rondani, 1860

New records: Učka Nature Park, Vela Učka, 19.VI.2012, E. Buenaventura, T. Pape, D. Whitmore leg. (3♂) (NHMD); Zmajevac, 9.VIII.2016, S. Krčmar leg. (1♂) (DBUO).

Literature records: Baranov (1928); Sljeme (Whitmore 2011).

European distribution: Albania, Andorra, Austria, Belarus, Bulgaria, Croatia, Czech Republic, Estonia, Finland, France (mainland), Germany, Hungary, Italy (mainland, Sicily), Latvia, Moldova, Poland, Romania, Russia (Central European Territory, South European Territory), Serbia, Slovakia, Spain (mainland), Sweden, Switzerland, Ukraine.

##### 96. Sarcophaga (Heteronychia) pseudobenaci (Baranov, 1942) (▲)

Records: Zmajevac, 25.VIII.2017, S. Krčmar leg. (2♂) (DBUO); same locality, 11.IX.2017, S. Krčmar leg. (1♂) (DBUO).

Distribution: Bulgaria, Croatia, Greece (mainland), Serbia.

##### 97. Sarcophaga (Heteronychia) pumila Meigen, 1826

Literature records: Krka Falls (Strobl 1900); Zagreb; Novi Marof (Baranov 1942; Whitmore et al. 2013); Pape (2004).

Distribution: Austria, Belgium, Bulgaria, Croatia, Czech Republic, Denmark (mainland), Estonia, Finland, France (mainland), Germany, Hungary, Italy (mainland), Latvia, Lithuania, Norway (mainland), Poland, Romania, Russia (Central European Territory), Slovakia, Spain (mainland), Sweden, Switzerland, The Netherlands, Ukraine, United Kingdom.

##### 98. Sarcophaga (Heteronychia) rondaniana (Rohdendorf, 1937)

Literature records: Dalmatia (Böttcher 1913, as *arvorum* Rondani); Krapina; Mraclin; Pag Is.; Zagreb (Baranov 1942, as *arvorum*); Povolný and Verves (1997); Pape (2004); Labin (Whitmore et al. 2013).

European distribution: Austria, Bulgaria, Croatia, Czech Republic, France (mainland), Germany, Greece (mainland), Hungary, Italy (mainland), Macedonia, Romania, Serbia, Slovakia, Spain (mainland), The Netherlands, Ukraine.

##### 99. Sarcophaga (Heteronychia) schineri Bezzi, 1891

New records: Baške Oštarije, 13.VI.2012, E. Buenaventura, T. Pape, D. Whitmore leg. (2♂) (NHMD); Sertić Poljana, 17.VI.2012, E. Buenaventura, T. Pape, D. Whitmore leg. (1♂) (NHMD); Zmajevac, 26.VII.2017, S. Krčmar leg. (1♂) (DBUO); Slatinski Drenovac, 17.VIII.2017, S. Krčmar leg. (1♂) (DBUO).

Literature records: Zagreb, Podsused; Medvednica, Sljeme (Baranov 1942); Zagreb (Strukan 1964); Pape (2004); Zagreb, Podsused; Velika Kapela Mts, Vrh Kapele (Whitmore et al. 2013).

European distribution: Albania, Austria, Bulgaria, Croatia, Czech Republic, France (mainland), Germany, Greece (mainland), Hungary, Italy (mainland, Sicily), Poland, Romania, Russia (South European Territory), Serbia, Slovakia, Switzerland, Ukraine.

##### 100. Sarcophaga (Heteronychia) vagans Meigen, 1826

New records: Podoštra, 15.VI.2012, E. Buenaventura, T. Pape, D. Whitmore leg. (2♂) (NHMD).

Literature records: Dubrovnik (Strobl 1900); Mraclin; Pag Is. (Baranov 1938, as *cruenta* Pandellé); Mraclin; Pag Is.; Samobor; Zagreb (Baranov 1942, as *frenata* Pandellé, *cruentata* Pandellé and *anastrenua* Baranov); Zagreb (Whitmore 2011).

European distribution: Andorra, Austria, Belarus, Belgium, Bulgaria, Croatia, Czech Republic, Denmark (mainland), Estonia, Finland, France (mainland), Germany, Hungary, Ireland, Italy (mainland), Latvia, Moldova, Norway (mainland), Poland, Romania, Russia (Central European Territory, North European Territory, South European Territory), Serbia, Slovakia, Spain (mainland), Sweden, Switzerland, The Netherlands, Ukraine, United Kingdom.

##### 101. Sarcophaga (Heteronychia) vicina Macquart, 1835

New records: Sušanj Cesarički, 13.VI.2012, E. Buenaventura, T. Pape, D. Whitmore leg. (1♂) (NHMD); Podoštra, 15.VI.2012, E. Buenaventura, T. Pape, D. Whitmore leg. (1♂) (NHMD); Plitvička jezera National Park, Turčić, 16.VI.2012, T. Pape et al. leg. (1♂) (NHMD); Begovo Razdolje, nr Bijele Stijene, 18.VI.2012, T. Pape et al. leg. (1♀, 1♂) (NHMD).

Literature records: Zagreb (Baranov 1942, as *ebrachiata* Pandellé); Whitmore (2010).

European distribution: Austria, Bosnia and Herzegovina, Bulgaria, Croatia, Czech Republic, Finland, France (mainland, Corsica), Germany, Greece (mainland), Hungary, Ireland, Italy (mainland), Norway (mainland), Poland, Russia (South European Territory), Slovakia, Spain (mainland), Sweden, Switzerland, Ukraine, United Kingdom.

##### 102. Sarcophaga (Heteronychia) sp.

Records: Skrad, 31.V.2017, S. Krčmar leg. (2♂) (DBUO).

Remarks: These two males belong to an undescribed species, probably ascribable to the *ancilla*-group, characterised by lateral styli conspicuously enlarged apically. They may be conspecific with specimens misidentified as *ancilla* by Povolný (1996, fig. 19) and Povolný and Verves (1997, fig. 177).

##### 103. Sarcophaga (Kramerea) schuetzei Kramer, 1909

Literature records: Zagreb (Baranov 1942); Oprić (Sisojević et al. 1989); Xue et al. (2011).

European distribution: Austria, Belarus, Bulgaria, Croatia, Czech Republic, France (mainland), Germany, Hungary, Italy (mainland), Poland, Russia (Central European Territory, North European Territory, South European Territory), Slovakia, Switzerland, Ukraine.

##### 104. Sarcophaga (Krameromyia) anaces Walker, 1849

New records: Novi Grad, 27–31.V.1958, R.L. Coe leg. (3♂) (NHMUK); Zmajevac, 30.IV.2017, S. Krčmar leg. (1♂) (DBUO); same locality, 11.09.2017, S. Krčmar leg. (1♂) (DBUO).

Literature records: Hvar; Solin; Šibenik (Strobl 1900, as *setipennis* Rondani); Krapina; Pag Is.; Zagreb, Podsused (Baranov 1942, as *setipennis*); Omiš, Zakučac (Sisojević et al. 1989); Pape (2004).

European distribution: Austria, Belgium, Bulgaria, Croatia, Czech Republic, France (mainland), Germany, Hungary, Italy (mainland), Poland, Slovakia, Spain (mainland), Switzerland, The Netherlands, United Kingdom.

##### 105. Sarcophaga (Latistyla) czernyi Böttcher, 1912

Literature records: Dubrovnik (Böttcher 1912); Dubrovnik; Otočac (Baranov 1942); Velika Paklenica (Strukan 1970); Pape (1996, 2004).

European distribution: Croatia, Greece (mainland).

##### 106. Sarcophaga (Liopygia) argyrostoma (Robineau-Desvoidy, 1830)

New records: Zmajevac, 25.VIII.2017, S. Krčmar leg. (1♂) (DBUO).

Literature records: Dalmatia (Böttcher 1913, as *falculata* Pandellé); Pag Is., Metajna (Baranov 1940, as *barbata* Thomson); Zagreb; Samobor; Krapina; Pag Is.; Krk Is.; Split; Dubrovnik (Baranov 1942, as *falculata*); Velika Paklenica; Premuda (Strukan 1964); Biokovo Mts (Povolný and Znojil 1998, 1999); Krk Is. (Povolný and Znojil 1998); Podgora (Povolný and Znojil 1994, 1998, 1999); Pape (2004).

European distribution: Albania, Austria, Belgium, Bulgaria, Croatia, Cyprus, Czech Republic, Denmark (mainland), France (mainland, Corsica), Germany, Greece (mainland), Hungary, Italy (mainland, Sardinia, Sicily), Macedonia, Moldova, Poland, Portugal (mainland, Madeira Is.), Romania, Russia (Central European Territory, South European Territory), Serbia, Slovakia, Spain (mainland, Canary Is.), Switzerland, The Netherlands, Ukraine, United Kingdom.

##### 107. Sarcophaga (Liopygia) crassipalpis Macquart, 1839

New records: Hvar Is., Dubovica, 12.VII.2004, D. Whitmore leg. (4♂) (DW); Tribanj-Krušćica, 14.VI.2012, E. Buenaventura, T. Pape, D. Whitmore leg. (2♂) (NHMD); Zmajevac, 4.VII.2014, S. Krčmar leg. (1♂) (DBUO); Modro Oko, 18.VII.2014, S. Krčmar leg. (3♂) (DBUO); Biograd, 18.VIII.2014, S. Krčmar leg. (1♂) (DBUO); same locality, 19.VIII.2014, S. Krčmar leg. (3♂) (DBUO); same locality, 20.VIII.2014, S. Krčmar leg. (1♂) (DBUO).

Literature records: Dalmatia (Schiner 1862, as *dalmatina* Schiner; Böttcher 1913, as *securifera* Villeneuve); Samobor (Baranov 1942, as *securifera*); Trogir (Strukan 1964, 1970); Biokovo Mts; Podgora (Povolný and Znojil 1994, 1998, 1999); Pape (1996); Krk Is. (Povolný and Znojil 1998); Pape (2004).

European distribution: Albania, Bulgaria, Croatia, Cyprus, Czech Republic, France (mainland, Corsica), Germany, Greece (mainland), Hungary, Italy (mainland, Sardinia, Sicily), Malta, Moldova, Portugal (mainland, Madeira Is.), Romania, Russia (Central European Territory, South European Territory), Serbia, Slovakia, Spain (mainland, Canary Is.).

##### 108. Sarcophaga (Liosarcophaga) aegyptica Salem, 1935

New records: Zmajevac, 11.IX.2017, S. Krčmar leg. (1♂) (DBUO).

Literature records: Oprić (Sisojević et al. 1989).

European distribution: Albania, Bulgaria, Croatia, Czech Republic, France (mainland), Hungary, Italy (mainland), Moldova, Romania, Russia (Central European Territory, South European Territory), Slovakia, Ukraine.

##### 109. Sarcophaga (Liosarcophaga) dux Thomson, 1869

New records: Korčula, 22–27.V.1955, R.L. Coe leg. (2♂) (NHMUK); Novi Grad, 27–31.V.1958, R.L. Coe leg. (1♂) (NHMUK); Rudelić Draga, 14.VI.2012, E. Buenaventura, T. Pape, D. Whitmore leg. (1♂) (NHMD).

Literature records: Baranov (1928, 1929, as *exuberans* Pandellé); Dalmatia (Baranov 1929, as *exuberans* var. setosa Baranov); Pag Is., Metajna (Baranov 1940, as *exuberans*); Dubrovnik (Baranov 1942, as *exuberans*); Premuda; Velika Paklenica (Strukan 1964, 1970, as *exuberans*); Podgora (Povolný and Znojil 1994, 1998, 1999); Pape (1996, 2004).

European distribution: Albania, Bulgaria, Croatia, Cyprus, France (mainland, Corsica), Greece (mainland), Italy (mainland, Sardinia, Sicily), Malta, Romania, Serbia, Spain (mainland, Canary Is.), Ukraine.

##### 110. Sarcophaga (Liosarcophaga) emdeni (Rohdendorf, 1969)

New records: Učka Nature Park, Vela Učka, 19.VI.2012, E. Buenaventura, T. Pape, D. Whitmore leg. (1♂) (NHMD); Skrad, 11.VI.2016, S. Krčmar leg. (1♂) (DBUO); Zmajevac, 29.V.2016, S. Krčmar leg. (2♂) (DBUO); same locality, 14.V.2017, S. Krčmar leg. (4♂) (DBUO); same locality, 26.VII.2017, S. Krčmar leg. (2♂) (DBUO); same locality, 10.VIII.2017, S. Krčmar leg. (1♂) (DBUO); same locality, 11.IX.2017, S. Krčmar leg. (2♂) (DBUO); Slatinski Drenovac, 17.VIII.2017, S. Krčmar leg. (1♂) (DBUO).

Literature records: Baranov (1928, 1929, as *teretirostris* Pandellé); Zagreb; Dalmatia (Baranov 1942, as *teretirostris*); Zagreb (Strukan 1970, as *teretirostris*); Oprić (Sisojević et al. 1989); Podgora (Povolný and Znojil 1998, 1999); Pape (2004).

European distribution: Austria, Bulgaria, Croatia, Czech Republic, Denmark (mainland), Estonia, Finland, Germany, Greece (mainland), Hungary, Italy (mainland), Norway (mainland), Poland, Romania, Russia (Central European Territory, South European Territory), Slovakia, Sweden, Switzerland, Ukraine.

##### 111. Sarcophaga (Liosarcophaga) harpax Pandellé, 1896

Literature records: Zagreb (Baranov 1929, 1942); Velika Paklenica (Strukan 1970); Sisojević et al. (1989); Podgora (Povolný and Znojil 1998, 1999); Pape (2004).

European distribution: Austria, Belarus, Bulgaria, Croatia, Czech Republic, France (mainland), Germany, Hungary, Italy (mainland), Moldova, Poland, Romania, Russia (Central European Territory, North European Territory, South European Territory), Serbia, Slovakia, The Netherlands, Ukraine.

Remarks: We here record this species from Italy for the first time (Perugia, Gubbio, Scritto, 43°14'42.98"N, 12°32'48.82"E, 475 m, 15.VIII.2007, D. Whitmore leg., 1♂, DW).

##### 112. Sarcophaga (Liosarcophaga) jacobsoni (Rohdendorf, 1937)

New records: Novi Grad, 27–31.V.1958, R.L. Coe leg. (1♂) (NHMUK).

Literature records: Pag Is. (Baranov 1942); Bačinci; Premuda; Pula; Trogir (Strukan 1964, 1970); Metković, Šibanica (Rucner 1994); Podgora (Povolný and Znojil 1998, 1999); Pape (2004).

European distribution: Albania, Bulgaria, Croatia, Cyprus, Denmark (mainland), France (mainland, Corsica), Germany, Greece (mainland), Hungary, Ireland, Italy (mainland, Sardinia), Moldova, Romania, Russia (Central European Territory, South European Territory), Slovakia, Spain (mainland, Canary Is.), Ukraine, United Kingdom.

Remarks: This species was not listed for Sardinia by Pape (2004), but has been known from the island at least since Povolný (1997).

##### 113. Sarcophaga (Liosarcophaga) marshalli Parker, 1923

New records: Novi Grad, 27–31.V.1958, R.L. Coe leg. (3♂) (NHMUK); Brač Is., Bol, 4.VIII.2004, D. Whitmore leg. (1♂) (DW).

Literature records: Premuda; Trogir (Strukan 1970, as *kovatschevitchi* Strukan); Pag Is. (Povolný 1999); Pape (2004).

European distribution: Croatia, France (mainland), Italy (mainland, Sicily), Malta, Spain (mainland).

Remarks: *Parasarcophagakovatschevitchi* Strukan, 1970 was described from one male collected in Trogir (15.VIII.1960) and fifteen males collected on the island of Premuda [18–19.VIII.1961 (4♂); 8–19.VIII.1963 (11♂)]. Based on the description and original drawings of the male terminalia of the new species, we propose *Parasarcophagakovatschevitchi* Strukan, 1970 as a junior synonym of Sarcophaga (Liosarcophaga) marshalli Parker, 1923, syn. nov.

##### 114. Sarcophaga (Liosarcophaga) portschinskyi (Rohdendorf, 1937)

New records: Sveti Juraj, 12.VI.2012, E. Buenaventura, T. Pape, D. Whitmore leg. (2♂) (NHMD); Modro Oko, 18.VII.2014, S. Krčmar leg. (1♂) (DBUO); Podrujnica, 18.VII.2014, S. Krčmar leg. (1♂) (DBUO); Vriještica, 3.VII.2015, S. Krčmar leg. (9♂) (DBUO); Zmajevac, 20.VII.2014, S. Krčmar leg. (1♀) (DBUO).

Literature records: Trogir; Premuda; Velika Paklenica (Strukan 1964, 1970); Podgora (Povolný and Znojil 1994, 1998, 1999); Pape (2004).

European distribution: Albania, Andorra, Austria, Belarus, Belgium, Bulgaria, Croatia, Czech Republic, Denmark (mainland), Estonia, Finland, France (mainland, Corsica), Germany, Greece (mainland), Hungary, Ireland, Italy (mainland, Sardinia, Sicily), Malta, Moldova, Norway (mainland), Poland, Romania, Russia (Central European Territory, South European Territory), Slovakia, Sweden, Switzerland, The Netherlands, Ukraine.

##### 115. Sarcophaga (Liosarcophaga) tibialis Macquart, 1851

New records: Tribanj-Krušćica, 14.VI.2012, E. Buenaventura, T. Pape, D. Whitmore leg. (1♂) (NHMD); Crikvenica, 5.VI.2014, S. Krčmar leg. (2♂) (DBUO); Desne, 17.VII.2014, S. Krčmar leg. (1♂) (DBUO); Modro Oko, 18.VII.2014, S. Krčmar leg. (2♂) (DBUO); Podrujnica, 18.VII.2014, S. Krčmar leg. (2♂) (DBUO); Biograd, 20.VIII.2014, S. Krčmar leg. (2♂) (DBUO); Krk Is., Punat, 1.VI.2017, S. Krčmar leg. (6♂) (DBUO); same locality, 7.VI.2017, S. Krčmar leg. (1♂) (DBUO).

Literature records: Pag Is., Metajna (Baranov 1940, as *beckeri* Villeneuve); Pag Is.; Krk Is. (Baranov 1942, as *beckeri*); Premuda; Velika Paklenica; Zadar (Strukan 1964, 1970); Podgora (Povolný and Znojil 1994, 1998, 1999); Pape (2004).

European distribution: Bulgaria, Croatia, Czech Republic, France (mainland, Corsica), Greece (mainland), Italy (mainland, Sardinia, Sicily), Portugal (Madeira Is.), Malta, Spain (mainland, Canary Is.).

Remarks: This species was not listed for Bulgaria by Pape (2004), but has been known from the country at least since Drenski (1957).

##### 116. Sarcophaga (Liosarcophaga) tuberosa Pandellé, 1896

New records: Podoštra, 15.VI.2012, E. Buenaventura, T. Pape, D. Whitmore leg. (1♂) (NHMD); Vriještica, 3.VII.2015, S. Krčmar leg. (1♂) (DBUO).

Literature records: Kostrena (Langhoffer 1920); Baranov (1928); Krapina; Zagreb (Baranov 1942); Otočac, Metla (Rucner 1994).

European distribution: Austria, Belgium, Bulgaria, Croatia, Czech Republic, France (mainland, Corsica), Germany, Greece (mainland), Hungary, Italy (mainland, Sardinia), Poland, Romania, Russia (Central European Territory, South European Territory), Serbia, Slovakia, Spain (mainland), Switzerland, The Netherlands, Ukraine.

##### 117. Sarcophaga (Mehria) nemoralis Kramer, 1908

New records: Podoštra, 15.VI.2012, E. Buenaventura, T. Pape, D. Whitmore leg. (1♂) (NHMD); Plitvička jezera National Park, Turčić, 16.VI.2012, E. Buenaventura, T. Pape, D. Whitmore leg. (2♂) (NHMD).

Literature records: Zagreb (Baranov 1942); Pape (2004).

European distribution: Austria, Bulgaria, Croatia, Czech Republic, Finland, Germany, Hungary, Poland, Romania, Russia (Central European Territory, South European Territory), Slovakia, Sweden, Switzerland, The Netherlands, Ukraine.

##### 118. Sarcophaga (Mehria) sexpunctata (Fabricius, 1805)

New records: Baške Oštarije, 13.VI.2012, E. Buenaventura, T. Pape, D. Whitmore leg. (1♂) (NHMD); Podoštra, 15.VI.2012, E. Buenaventura, T. Pape, D. Whitmore leg. (2♂) (NHMD).

Literature records: Samobor; Stubičke Toplice; Zagreb (Baranov 1942, as *clathrata* Meigen); Pape (2004).

European distribution: Austria, Belgium, Bulgaria, Croatia, Czech Republic, Denmark (mainland), Finland, France (mainland), Germany, Greece (mainland), Hungary, Ireland, Italy (mainland), Norway (mainland), Poland, Romania, Russia (Central European Territory, South European Territory), Slovakia, Spain (mainland, Canary Is.), Sweden, Switzerland, The Netherlands, Ukraine, United Kingdom.

##### 119. Sarcophaga (Mimarhopocnemis) granulata Kramer, 1908

Literature records: Zagreb; Požega; Krapina (Baranov 1942); Bačinci (Strukan 1964); Pape (2004).

Distribution: Austria, Belgium, Bulgaria, Croatia, Czech Republic, Finland, France (mainland), Germany, Hungary, Italy (mainland), Poland, Russia (Central European Territory), Slovakia, Spain (mainland), Ukraine.

##### 120. Sarcophaga (Myorhina) lunigera Böttcher, 1914 (▲)

Records: Plitvička jezera National Park, Turčić, 16.VI.2012, E. Buenaventura, T. Pape, D. Whitmore leg. (3♂) (NHMD); Begovo Razdolje, nr Bijele Stijene, 18.VI.2012, E. Buenaventura, T. Pape, D. Whitmore leg. (2♂) (NHMD); Učka Nature Park, Vela Učka, 19.VI.2012, E. Buenaventura, T. Pape, D. Whitmore leg. (1♂) (NHMD).

European distribution: Austria, Bosnia and Herzegovina, Croatia, Czech Republic, France (mainland), Germany, Poland, Romania, Russia (South European Territory), Serbia, Slovakia, Switzerland, Ukraine.

##### 121. Sarcophaga (Myorhina) nigriventris Meigen, 1826

New records: Sušanj Cesarički, 13.VI.2012, E. Buenaventura, T. Pape, D. Whitmore leg. (1♂) (NHMD); Plitvička jezera National Park, Turčić, 16.VI.2012, E. Buenaventura, T. Pape, D. Whitmore leg. (12♂) (NHMD); Zmajevac, 14.V.2017, S. Krčmar leg. (1♂) (DBUO).

Literature records: Zadar (Strobl 1904); Bakar; Senj; Zagreb (Langhoffer 1920); Baranov (1928); Delnice; Otočac; Pag Is.; Samobor; Zagreb (Baranov 1942); Biokovo Mts (Povolný and Znojil 1998, 1999); Pape (2004).

European distribution: Albania, Andorra, Austria, Belgium, Bulgaria, Croatia, Cyprus, Czech Republic, Denmark (mainland), France (mainland, Corsica), Germany, Greece (mainland), Hungary, Ireland, Italy (mainland, Sardinia, Sicily), Malta, Poland, Romania, Russia (South European Territory), Serbia, Slovakia, Spain (mainland), Switzerland, The Netherlands, Ukraine, United Kingdom.

##### 122. Sarcophaga (Myorhina) pandifera Blackith & Pape, 1999

Literature records: Sljeme (Baranov 1942, as *discifera* Pandellé); Pape (2004).

Distribution: Austria, Croatia, Czech Republic, France (mainland), Germany, Italy (mainland), Poland, Romania, Slovakia, Switzerland, Ukraine.

##### 123. Sarcophaga (Myorhina) socrus Rondani, 1860

New records: Sveti Juraj, 12.VI.2012, E. Buenaventura, T. Pape, D. Whitmore leg. (1♂) (NHMD); Sušanj Cesarički, 13.VI.2012, E. Buenaventura, T. Pape, D. Whitmore leg. (1♂) (NHMD).

Literature records: Krapina; Mrzla Vodica (Baranov 1942, as *rostrata* Pandellé); Otočac, Metla (Rucner 1994, as *rostrata*); Biokovo Mts; Podgora (Povolný and Znojil 1998, 1999); Pape (2004).

Distribution: Albania, Andorra, Austria, Bulgaria, Croatia, Czech Republic, Estonia, Finland, France (mainland), Germany, Greece (mainland), Hungary, Italy (mainland, Sicily), Poland, Russia (Central European Territory, South European Territory), Slovakia, Switzerland, Ukraine.

##### 124. Sarcophaga (Myorhina) soror Rondani, 1860

New records: Sušanj Cesarički, 13.VI.2012, E. Buenaventura, T. Pape, D. Whitmore leg. (2♂) (NHMD); Zmajevac, 30.IV.2017, S. Krčmar leg. (1♂) (DBUO).

Literature records: Dubrovnik (Strobl 1900); Krapina; Otočac; Samobor; Zagreb; Sljeme (Baranov 1942); Biokovo Mts; Podgora (Povolný and Znojil 1998, 1999); Pape (2004).

European distribution: Austria, Bulgaria, Croatia, Czech Republic, Denmark (mainland), Estonia, France (mainland), Germany, Hungary, Ireland, Italy (mainland, Sicily), Norway (mainland), Poland, Romania, Russia (Central European Territory, South European Territory), Slovakia, Spain (mainland, Canary Is.), Sweden, Switzerland, Ukraine, United Kingdom.

##### 125. Sarcophaga (Pandelleana) protuberans Pandellé, 1896

New records: Korčula, 22–27.V.1955, R.L. Coe leg. (3♂) (NHMUK); Sveti Juraj, 12.VI.2012, E. Buenaventura, T. Pape, D. Whitmore leg. (1♂) (NHMD); Sušanj Cesarički, 13.VI.2012, E. Buenaventura, T. Pape, D. Whitmore leg. (2♂) (NHMD).

Literature records: Pag Is.; Sljeme; Zagreb (Baranov 1942); Biokovo Mts; Podgora (Povolný and Znojil 1998, 1999); Pape (2004).

European distribution: Austria, Bulgaria, Croatia, Czech Republic, France (mainland, Corsica), Germany, Hungary, Italy (mainland, Sicily), Moldova, Poland, Romania, Russia (Central European Territory, South European Territory), Serbia, Slovakia, Spain (mainland), Switzerland, The Netherlands, Ukraine.

##### 126. Sarcophaga (Pandelleisca) similis Meade, 1876

New records: Komin, 16.VII.2014, S. Krčmar leg. (4♂) (DBUO); Badžula, 18.VII.2014, S. Krčmar leg. (2♂) (DBUO); Vid 17.VII.2014, S. Krčmar leg. (1♂) (DBUO); Desne, 17.VII.2014, S. Krčmar leg. (1♂) (DBUO); Modro Oko, 18.VII.2014, S. Krčmar leg. (1♂) (DBUO); Skrad, 11.VI.2016, S. Krčmar leg. (1♂) (DBUO); same locality, 31.V.2017, S. Krčmar leg. (4♂) (DBUO); Zmajevac, 25.VIII.2017, S. Krčmar leg. (1♂) (DBUO).

Literature records: Klana (Langhoffer 1920); Baranov (1928); Zagreb (Baranov 1942); Zagreb, Maksimir (Strukan 1964, 1970); Oprić (Sisojević et al. 1989); Pape (2004).

European distribution: Albania, Austria, Belarus, Belgium, Bulgaria, Croatia, Czech Republic, Estonia, Finland, France (mainland, Corsica), Germany, Hungary, Italy (mainland), Latvia, Moldova, Norway (mainland), Poland, Romania, Russia (Central European Territory, South European Territory), Slovakia, Sweden, Switzerland, The Netherlands, Ukraine, United Kingdom.

##### 127. Sarcophaga (Parasarcophaga) albiceps Meigen, 1826

New records: Novi Grad, 27–31.V.1958, R.L. Coe leg. (3♂) (NHMUK); Crikvenica, 5.VI.2014, S. Krčmar leg. (1♂) (DBUO); Zmajevac, 27.VI.2014, S. Krčmar leg. (2♂) (DBUO); same locality, 29.VI.2014, S. Krčmar leg. (4♂) (DBUO); same locality, 2.VII.2014, S. Krčmar leg. (2♂) (DBUO); same locality, 3.VII.2014, S. Krčmar leg. (3♂) (DBUO); same locality, 4.VII.2014, S. Krčmar leg. (16♂) (DBUO); same locality, 26.VIII.2014, S. Krčmar leg. (1♂) (DBUO); same locality, 15.VII.2015, S. Krčmar leg. (9♂) (DBUO); same locality, 9.VIII.2016, S. Krčmar leg. (2♂) (DBUO); same locality, 18.VII.2017, S. Krčmar leg. (1♂) (DBUO); same locality, 26.VII.2017, S. Krčmar leg. (1♂) (DBUO); same locality, 10.VIII.2017, S. Krčmar leg. (2♂) (DBUO); same locality, 25.VIII.2017, S. Krčmar leg. (3♂) (DBUO); same locality, 11.IX.2017, S. Krčmar leg. (6♂) (DBUO); Desne, 17.VII.2014, S. Krčmar leg. (6♂) (DBUO); same locality, 17.VII.2015, S. Krčmar leg. (1♂) (DBUO); Modro Oko, 18.VII.2014, S. Krčmar leg. (2♂) (DBUO); Biograd, 19.VIII.2014, S. Krčmar leg. (4♂) (DBUO); Skrad, 8.VI.2017, S. Krčmar leg. (1♂) (DBUO).

Literature records: Rijeka (Strobl 1893); Dubrovnik (Strobl 1900, as *privigna* Rondani); Delnice; Lokve (Langhoffer 1920); Baranov (1928); Delnice; Krapina; Zagreb (Baranov 1942); Trogir; Premuda; Velika Paklenica; Zadar (Strukan 1970); Oprić; Vozilići (Sisojević et al. 1989); Našice, Prkos; Samoborsko gorje, Čudomerščak; Ston, Česvinica (Rucner 1994); Podgora (Povolný and Znojil 1994, 1998, 1999); Pape (2004).

European distribution: Albania, Austria, Belarus, Belgium, Bulgaria, Croatia, Czech Republic, Finland, France (mainland), Germany, Greece (mainland), Hungary, Italy (mainland, Sicily), Latvia, Moldova, Norway (mainland), Poland, Romania, Russia (Central European Territory, North European Territory, South European Territory), Serbia, Slovakia, Spain (mainland), Sweden, Switzerland, The Netherlands, Ukraine, United Kingdom.

##### 128. Sarcophaga (Phytosarcophaga) destructor Malloch, 1929

Literature records: Trogir (Strukan 1964, 1968, as *destructrix* Malloch).

European distribution: Croatia, Cyprus, France (mainland), Greece (mainland), Italy (mainland), Spain (mainland).

##### 129. Sarcophaga (Robineauella) caerulescens Zetterstedt, 1838

New records: Podoštra, 15.VI.2012, E. Buenaventura, T. Pape, D. Whitmore leg. (2♂) (NHMD); Sunger, 6.VI.2014, S. Krčmar leg. (1♂) (DBUO); Zmajevac, 4.VII.2014, S. Krčmar leg. (1♂) (DBUO).

Literature records: Samobor; Skrad; Stubičke Toplice; Zagreb (Baranov 1942, as *scoparia* Pandellé); Samoborsko gorje, Čudomerščak (Rucner 1994, as *scoparia*); Pape (2004).

European distribution: Austria, Belarus, Belgium, Bulgaria, Croatia, Czech Republic, Denmark (mainland), Estonia, Finland, France (mainland), Germany, Hungary, Italy (mainland, Sicily), Latvia, Norway (mainland), Poland, Romania, Russia (Central European Territory, North European Territory, South European Territory), Slovakia, Spain (mainland), Sweden, Switzerland, Ukraine, United Kingdom.

##### 130. Sarcophaga (Rosellea) aratrix Pandellé, 1896

New records: Podoštra, 15.VI.2012, E. Buenaventura, T. Pape, D. Whitmore leg. (1♂) (NHMD); Sunger, 6.VI.2014, S. Krčmar leg. (2♂) (DBUO); same locality, 14.VI.2016, S. Krčmar leg. (1♂) (DBUO); Zmajevac, 27.VI.2014, S. Krčmar leg. (1♂) (DBUO); same locality, 2.VII.2014, S. Krčmar leg. (1♂) (DBUO); same locality, 4.VII.2014, S. Krčmar leg. (1♂) (DBUO); same locality, 30.IV.2017, S. Krčmar leg. (1♂) (DBUO); same locality, 14.V.2017, S. Krčmar leg. (2♂) (DBUO); Modro oko, 18.VII.2014, S. Krčmar leg. (2♂) (DBUO); Skrad, 11.VI.2016, S. Krčmar leg. (3♂) (DBUO); same locality, 31.V.2017, S. Krčmar, leg. (3♂) (DBUO); Kamenac, 23.VII.2016, S. Krčmar leg. (1♂) (DBUO); Krk Is., Punat, 1.VI.2017, S. Krčmar leg. (5♂) (DBUO).

Literature records: Crikvenica; Zagreb (Langhoffer 1920); Baranov (1928); Krapina; Mraclin; Zagreb (Baranov 1942); Kuzmin (Strukan 1964, 1970); Oprić; Vozilići (Sisojević et al. 1989); Vinica; Petrova gora, Brđani; Livade; Otočac, Šumečica; Otočac, Metla (Rucner 1994); Pape (2004).

European distribution: Albania, Austria, Belarus, Belgium, Bulgaria, Croatia, Czech Republic, Denmark (mainland), Estonia, Finland, France (mainland, Corsica), Germany, Hungary, Ireland, Italy (mainland, Sicily), Latvia, Lithuania, Moldova, Norway (mainland), Poland, Romania, Russia (Central European Territory, South European Territory), Serbia, Slovakia, Spain (mainland), Sweden, Switzerland, The Netherlands, Ukraine, United Kingdom.

##### 131. Sarcophaga (Sarcophaga) adriatica Böttcher, 1913

New records: Sveti Juraj, 12.VI.2012, E. Buenaventura, T. Pape, D. Whitmore leg. (5♂) (NHMD); Tribanj-Krušćica, 14.VI.2012, E. Buenaventura, T. Pape, D. Whitmore leg. (3♂) (NHMD).

Literature records: Dalmatia; Istria (Böttcher 1913, as *vicina* var. adriatica); Pag Is. (Baranov 1942); Premuda (Strukan 1964, 1967); Pape (1996); Krk Is. (Povolný and Znojil 1998); Pape (2004).

Distribution: Croatia, Serbia.

##### 132. Sarcophaga (Sarcophaga) baranoffi Rohdendorf, 1937

New records: Samobor, 25.V.1930, N. Baranov leg. (2♂) (ZMHB); Podoštra, 15.VI.2012, E. Buenaventura, T. Pape, D. Whitmore leg. (2♂) (NHMD); Krk Is., Punat, 4.VI.2014, S. Krčmar leg. (1♂) (DBUO); Drivenik, 5.VI.2014, S. Krčmar leg. (1♂) (DBUO); Bjelolasica Mts, 2.VI.2015, S. Krčmar leg. (3♂) (DBUO); Kamenac, 23.VII.2016, S. Krčmar leg. (1♂) (DBUO); Zmajevac, 9.VIII.2016, S. Krčmar leg. (1♂) (DBUO); same locality, 28.VIII.2016, S. Krčmar leg. (2♂) (DBUO); same locality, 30.IX.2016, S. Krčmar leg. (1♂) (DBUO); same locality, 30.IV.2017, S. Krčmar leg. (2♂) (DBUO); same locality, 14.V.2017, S. Krčmar leg. (2♂) (DBUO); same locality, 18.VII.2017, S. Krčmar leg. (1♂) (DBUO); same locality, 24.VII.2017, S. Krčmar leg. (2♂) (DBUO); same locality, 25.VIII.2017, S. Krčmar leg. (1♂) (DBUO); same locality, 11.IX.2017, S. Krčmar leg. (1♂) (DBUO); Skrad, 8.VI.2017, S. Krčmar leg. (1♂) (DBUO).

Literature records: Zagreb, Samobor (Rohdendorf 1937, as *subvicina* ssp. baranoffi); Delnice; Klek; Zagreb, Podsused; Požega; Samobor; Zagreb (Baranov 1942); Oprić (Sisojević et al. 1989); Pape (1996, 2004).

Distribution: Bulgaria, Croatia, Italy (mainland), Serbia, Slovenia.

##### 133. Sarcophaga (Sarcophaga) cf.bergi Rohdendorf, 1937

New records: Plitvička jezera National Park, Turčić, 16.VI.2012, E. Buenaventura, T. Pape, D. Whitmore leg. (1♂) (NHMD).

European distribution: Bulgaria, Croatia, Ukraine.

Remarks: The single male of this species examined from Croatia is morphologically very similar to *S.bergi*, but differs in certain features of the distiphallus and in the shape of the cercus in lateral view. It may belong to a different species, but more material and more detailed studies are needed to confirm this.

##### 134. Sarcophaga (Sarcophaga) carnaria (Linnaeus, 1758)

Literature records: Dubrovnik (Strobl 1900); Hvar; Zadar (Strobl 1904); Baranov (1928); Samobor (Baranov 1941b, 1942, as *subvicina* ssp. vulgaris Rohdendorf); Premuda; Zagreb, Maksimir (Strukan 1964); Pape (2004).

European distribution: Austria, Belarus, Belgium, Bulgaria, Croatia, Czech Republic, Denmark (mainland), Estonia, Finland, France (mainland), Germany, Hungary, Ireland, Italy (mainland), Luxemburg, Moldova, Norway (mainland), Poland, Romania, Russia (Central European Territory, North European Territory, South European Territory), Slovakia, Sweden, Switzerland, The Netherlands, Ukraine, United Kingdom.

##### 135. Sarcophaga (Sarcophaga) croatica Baranov, 1941

New records: Novi Grad, 27–31.V.1958, R.L. Coe leg. (1♂) (NHMUK); Rudelić Draga, 14.VI.2012, E. Buenaventura, T. Pape, D. Whitmore leg. (2♂) (NHMD); Sunger, 4.VI.2014, S. Krčmar leg. (2♂) (DBUO); same locality, 6.VI.2014, S. Krčmar leg. (5♂) (DBUO); same locality, 10.VI.2016, S. Krčmar leg. (1♂) (DBUO); same locality, 14.VI.2016, S. Krčmar leg. (2♂) (DBUO); Skrad, 1.VI.2015, S. Krčmar leg. (1♂) (DBUO); same locality, 31.V.2017, S. Krčmar leg. (10♂) (DBUO); same locality, 8.VI.2017, S. Krčmar leg. (6♂) (DBUO); Krk Is., 3.VI.2015, S. Krčmar leg. (1♂) (DBUO); Lokve, 2.VI.2017, S. Krčmar leg. (1♂) (DBUO); Zmajevac, 15.VII.2015, S. Krčmar leg. (1♂) (DBUO); same locality, 29.V.2016, S. Krčmar leg. (5♂) (DBUO); same locality, 9.VI.2016, S. Krčmar leg. (1♂) (DBUO); same locality, 9.VIII.2016, S. Krčmar leg. (11♂) (DBUO); same locality, 28.VIII.2016, S. Krčmar leg. (4♂) (DBUO); same locality, 30.IX.2016, S. Krčmar leg. (8♂) (DBUO); same locality, 30.IV.2017, S. Krčmar leg. (6♂) (DBUO); same locality, 14.V.2017, S. Krčmar leg. (14♂) (DBUO); same locality, 18.VII.2017, S. Krčmar leg. (24♂) (DBUO); same locality, 24.VII.2017, S. Krčmar leg. (18♂) (DBUO); same locality, 26.VII.2017, S. Krčmar leg. (17♂) (DBUO); same locality, 10.VIII.2017, S. Krčmar leg. (33♂) (DBUO); same locality, 11.IX.2017, S. Krčmar leg. (21♂) (DBUO); Krk Is., Punat, 13.VI.2016, S. Krčmar leg. (1♂) (DBUO); same locality, 1.VI.2017, S. Krčmar leg. (2♂) (DBUO); Kamenac, 23.VII.2016, S. Krčmar leg. (1♂) (DBUO); Čigoč, 5.VI.2017, S. Krčmar leg. (15♂) (DBUO); Orahovica, 4.VII.2017, S. Krčmar leg. (15♂) (DBUO); Slatinski Drenovac, 17.VIII.2017, S. Krčmar leg. (4♂) (DBUO).

Literature records: Zagreb; Samobor; Kaštel Sućurac; Pag Is. (Baranov 1941b, 1942, as *subvicina* ssp. croatica and *subvicina* ssp. novaki Baranov); Pape (2004).

Distribution: Croatia, Italy (mainland, Sicily).

Remarks: This species, which was the most abundant flesh fly in recent Croatian samples, is common and widespread also in Italy where it had previously been confused with the closely related *S.variegata* (Scopoli, 1763) (Whitmore et al., unpubl. data). We here formally record this species from Italy for the first time (238♂ from Liguria, Lombardy, Umbria, Lazio, Abruzzo, Campania, Basilicata, Calabria and Sicily, 1995–2015, DW and Museo di Zoologia, Sapienza Università di Roma). Records of *Sarcophagavariegata* from Sardinia (see Whitmore 2009a) may possibly also refer to *S.croatica*. Literature records of “*S.croatica*” from Corsica (e.g., Jordaens et al. 2013) refer to the Corsican endemic *S.matilei* Blackith, Richet, Pape and Andrei-Ruiz, 2001. Based on our recent examination of the holotype male of Sarcophagasubvicinassp.croatica Baranov, 1941 (NMNH) and of photographs of the holotype male of Sarcophagasubvicinassp.novaki Baranov, 1941 (NMNH), we propose Sarcophagasubvicinassp.novaki Baranov, 1941 as a junior synonym of Sarcophaga (Sarcophaga) croatica Baranov, 1941, syn. nov. This synonymy means that S. (S.) hennigi Lehrer, 1978 has not been recorded from Croatia. The previous listings of “*hennigi*” or “*novaki*” from Croatia by Verves (1986), Povolný (1996), Povolný and Verves (1997), Pape (1996, 2004) and Verves and Khrokalo (2014) were based on the erroneous synonymy with *S.novaki* proposed by Povolný and Verves (1987).

##### 136. Sarcophaga (Sarcophaga) lehmanni Müller, 1922

New records: Zmajevac, 2.VII.2014, S. Krčmar leg. (3♂) (DBUO); same locality, 4.VII.2014, S. Krčmar leg. (7♂) (DBUO); same locality, 26.VIII.2014, S. Krčmar leg. (1♂) (DBUO); same locality, 15.VII.2015, S. Krčmar leg. (4♂) (DBUO); same locality, 29.V.2016, S. Krčmar leg. (3♂) (DBUO); same locality, 9.VIII.2016, S. Krčmar leg. (5♂) (DBUO); same locality, 28.VIII.2016, S. Krčmar leg. (5♂) (DBUO); same locality, 30.IX.2016, S. Krčmar leg. (3♂) (DBUO); same locality, 30.IV.2017, S. Krčmar leg. (2♂) (DBUO); same locality, 14.V.2017, S. Krčmar leg. (13♂) (DBUO); same locality, 18.VII.2017, S. Krčmar leg. (1♂) (DBUO); same locality, 24.VII.2017, S. Krčmar leg. (7♂) (DBUO); same locality, 26.VII.2017, S. Krčmar leg. (24♂) (DBUO); same locality, 10.VIII.2017, S. Krčmar leg. (7♂) (DBUO); same locality, 25.VIII.2017, S. Krčmar leg. (4♂) (DBUO); same locality, 11.IX.2017, S. Krčmar leg. (1♂) (DBUO); Komin, 16.VII.2014, S. Krčmar leg. (3♂) (DBUO); same locality, 16.VII.2015, S. Krčmar leg. (1♂) (DBUO); Blace, 17.VII.2014, S. Krčmar leg. (1♂) (DBUO); Desne, 17.VII.2014, S. Krčmar leg. (2♂) (DBUO); Badžula, 18.VII.2014, S. Krčmar leg. (2♂) (DBUO); Modro Oko, 17.VII.2014, S. Krčmar leg. (1♂) (DBUO); same locality, 18.VII.2014, S. Krčmar leg. (2♂) (DBUO); Vid, 17.VII.2014, S. Krčmar leg. (1♂) (DBUO); Čigoč, 10.VI.2016, S. Krčmar leg. (9♂) (DBUO); same locality, 5.VI.2017, S. Krčmar leg. (15♂) (DBUO); Sunger, 6.VI.2014, S. Krčmar leg. (2♂) (DBUO); same locality, 10.VI.2016, S. Krčmar leg. (1♂) (DBUO); same locality, 14.VI.2016, S. Krčmar leg. (2♂) (DBUO); Krk Is., Punat, 13.VI.2016, S. Krčmar leg. (1♂) (DBUO); same locality, 1.VI.2017, S. Krčmar leg. (17♂) (DBUO); same locality, 7.VI.2017, S. Krčmar leg. (6♂) (DBUO); Kamenac, 23.VII.2016, S. Krčmar leg. (7♂) (DBUO); Skrad, 8.VI.2017, S. Krčmar leg. (1♂) (DBUO); Orahovica, 4.VII.2017, S. Krčmar leg. (6♂) (DBUO); Slatinski Drenovac, 17.VIII.2017, S. Krčmar leg. (2♂) (DBUO).

Literature records: Baranov (1942, as *carnaria* ssp. meridionalis Rohdendorf); Bačinci; Premuda; Zagreb, Maksimir (Strukan 1964, as *carnaria* ssp. meridionalis); Vozilići (Sisojević et al. 1989); Biokovo Mts (Povolný and Znojil 1998, as *lasiostyla* Macquart, misidentification).

European distribution: Albania, Andorra, Austria, Belarus, Belgium, Bulgaria, Cyprus, Croatia, Czech Republic, Denmark (mainland), Estonia, France (mainland), Germany, Greece (mainland), Hungary, Italy (mainland, Sardinia, Sicily), Latvia, Lithuania, Moldova, Norway (mainland), Poland, Romania, Russia (Central European Territory, South European Territory), Slovakia, Spain (mainland), Sweden, Switzerland, The Netherlands, Ukraine.

##### 137. Sarcophaga (Sarcophaga) moldavica Rohdendorf, 1937

Literature records: Pape (1996); Povolný and Verves (1997); Pape (2004).

Distribution: Croatia, Czech Republic, Hungary, Moldova, Poland, Romania, Slovakia, Ukraine.

##### 138. Sarcophaga (Sarcophaga) pagensis Baranov, 1939

New records: Sveti Juraj, 12.VI.2012, E. Buenaventura, T. Pape, D. Whitmore leg. (1♂) (NHMD); Rudelić Draga, 14.VI.2012, E. Buenaventura, T. Pape, D. Whitmore leg. (1♂) (NHMD).

Literature records: Pag Is. (Baranov 1939, 1942); Podgora (Povolný and Znojil 1994, 1998, 1999); Pape (1996); Biokovo Mts (Povolný and Znojil 1999); Pape (2004).

Distribution: Croatia, France (mainland), Greece (mainland).

##### 139. Sarcophaga (Sarcophaga) serbica Baranov, 1929

Literature records: Povolný and Verves (1997).

Distribution: Bulgaria, Croatia, Hungary, Romania, Russia (Central European Territory), Serbia, Slovakia, Ukraine.

##### 140. Sarcophaga (Sarcophaga) subvicina Rohdendorf, 1937

Literature records: Baranov (1930, as *vicina* Villeneuve); Krapina (Baranov 1942); Krndija, Londžica; Učka, Planik (Rucner 1994).

European distribution: Austria, Belarus, Belgium, Bulgaria, Croatia, Czech Republic, Denmark (mainland), Estonia, Finland, France (mainland, Corsica), Germany, Hungary, Ireland, Italy (mainland, Sicily), Latvia, Moldova, Poland, Romania, Russia (Central European Territory), Serbia, Slovakia, Spain (mainland), Sweden, Switzerland, The Netherlands, Ukraine, United Kingdom.

##### 141. Sarcophaga (Sarcophaga) ukrainica Rohdendorf, 1937

Literature records: Verves and Khrokalo (2014).

Distribution: Croatia, Hungary, Romania, Slovakia, Ukraine.

##### 142. Sarcophaga (Sarcophaga) variegata (Scopoli, 1763)

New records: Brušane, 15.VI.2012, E. Buenaventura, T. Pape, D. Whitmore leg. (2♂) (NHMD); Plitvička jezera National Park, Turčić, 16.VI.2012, E. Buenaventura, T. Pape, D. Whitmore leg. (2♂) (NHMD); Sušanj Cesarički, 13.VI.2012, E. Buenaventura, T. Pape, D. Whitmore leg. (3♂) (NHMD); Podoštra, 15.VI.2012, E. Buenaventura, T. Pape, D. Whitmore leg. (3♂) (NHMD).

Literature records: Pape (1996); Vozilići (Sisojević et al. 1989); Pape (2004).

European distribution: Albania, Andorra, Austria, Belarus, Belgium, Bulgaria, Croatia, Czech Republic, Denmark (mainland), Estonia, Finland, France (mainland), Germany, Greece (mainland), Hungary, Italy (mainland), Latvia, Lithuania, Luxemburg, Moldova, Norway (mainland), Poland, Portugal (mainland), Romania, Russia (Central European Territory, North European Territory, South European Territory), Serbia, Slovakia, Spain (mainland), Sweden, Switzerland, The Netherlands, Ukraine, United Kingdom.

##### 143. Sarcophaga (Sarcophaga) zumptiana Lehrer, 1959

Literature records: Krapina (Baranov 1942, as *subvicina* ssp. rohdendorfi Baranov); Pape (1996, 2004).

Distribution: Austria, Bosnia and Herzegovina, Croatia, Czech Republic, France (mainland), Hungary, Romania, Serbia, Slovakia, Ukraine.

##### 144. Sarcophaga (Sarcotachinella) sinuata Meigen, 1826

New Records: Čigoč, 10.VI.2016, S. Krčmar leg. (2♂) (DBUO); Zmajevac, 15.VII.2015, S. Krčmar leg. (2♂) (DBUO); same locality, 25.VIII.2017, S. Krčmar leg. (1♂) (DBUO).

Literature records: Zagreb (Langhoffer 1920); Baranov (1928); Krapina; Mraclin; Zagreb (Baranov 1942).

European distribution: Albania, Austria, Belarus, Belgium, Bulgaria, Croatia, Czech Republic, Denmark (mainland), Estonia, France (mainland), Germany, Hungary, Ireland, Italy (mainland), Latvia, Moldova, Norway (mainland), Poland, Romania, Russia (Central European Territory, North European Territory, South European Territory), Serbia, Slovakia, Spain (mainland), Sweden, The Netherlands, Ukraine, United Kingdom.

##### 145. Sarcophaga (Stackelbergeola) mehadiensis Böttcher, 1912 (▲)

Records: Rudelić Draga, 14.VI.2012, E. Buenaventura, T. Pape, D. Whitmore leg. (2♂) (NHMD).

European distribution: Croatia, Czech Republic, France (mainland), Greece (mainland), Romania.

##### 146. Sarcophaga (Thyrsocnema) incisilobata Pandellé, 1896

New records: Korčula, 22–27.V.1955, R.L. Coe leg. (3♂) (NHMUK); Plitvice, 4–10.VII.1955, R.L. Coe leg. (1♂) (NHMUK); Sveti Juraj, 12.VI.2012, E. Buenaventura, T. Pape, D. Whitmore leg. (2♂) (NHMD); Zmajevac, 4.VII.2014, S. Krčmar leg. (2♂) (DBUO); same locality, 15.VII.2015, S. Krčmar leg. (4♂) (DBUO); same locality, 14.V.2017, S. Krčmar leg. (1♂) (DBUO); same locality, 24.VII.2017, S. Krčmar leg. (1♂) (DBUO); Podrujnica, 18.VII.2014, S. Krčmar leg. (2♂) (DBUO); Biograd, 18.VIII.2014, S. Krčmar leg. (2♂) (DBUO); Krk Is., Punat, 1.VI.2017, S. Krčmar leg. (3♂) (DBUO); Skrad, 8.VI.2017, S. Krčmar leg. (1♂) (DBUO).

Literature records: Pag Is.; Samobor; Zagreb (Baranov 1942); Oprić (Sisojević et al. 1989); Metković, Šibanica; Samoborsko gorje, Palačnik; Otočac, Metla; Baške Oštarije, Velika Basača; Baške Oštarije, Filipov Kuk; Posedarje (Rucner 1994); Podgora (Povolný and Znojil 1994, 1998, 1999); Pape (2004).

European distribution: Albania, Andorra, Austria, Belarus, Belgium, Bulgaria, Croatia, Czech Republic, Denmark (mainland), Estonia, Finland, France (mainland), Germany, Greece (mainland), Hungary, Ireland, Italy (mainland), Lithuania, Moldova, Norway (mainland), Poland, Romania, Russia (Central European Territory, South European Territory), Serbia, Slovakia, Spain (mainland), Sweden, Switzerland, The Netherlands, Ukraine, United Kingdom.

Remarks: Records of the occurrence of *Sarcophagaincisilobata* in Sicily (e.g., Pape et al. 1995; Povolný 1999) refer to an undescribed species that also occurs in southern mainland Italy (Whitmore et al., unpubl. data).

##### 147. Sarcophaga (Thyrsocnema) platariae (Povolný, 1992)

New records: Komin, 16.VII.2014, S. Krčmar leg. (1♂) (DBUO).

Literature records: Pape (2004).

European distribution: Croatia, Greece (mainland).

Remarks: Povolný (1992) described *Thyrsocnemaplatariae* from a single male collected in Greece. He tentatively placed it in *Thyrsocnema* Enderlein based on the shape of the cercus and sternite 5, whereas Pape (1996: 418) later listed it as *incertae sedis* within *Sarcophaga*. A new genus was created by Lehrer (2000) for *Golaniaisraeliana* Lehrer, 2000. This species was synonymised with *Thyrsocnemaplatariae* by Pape (2004), who placed it in *Sarcophaga*, subgenus Golania Lehrer. Based on our study of recent material from Croatia (above) and Greece, we agree with Povolný’s original placement in *Thyrsocnema* and propose *Golania* Lehrer, 2000 as a junior synonym of *Thyrsocnema* Enderlein, 1928, syn. nov., at the subgeneric rank.

##### 148. Sarcophaga (Varirosellea) uliginosa Kramer, 1908

Literature records: Oprić (Sisojević et al. 1989); Pape (2004).

European distribution: Albania, Austria, Belarus, Bulgaria, Croatia, Czech Republic, Denmark (mainland), France (mainland), Germany, Greece (mainland), Hungary, Italy (mainland, Sardinia), Moldova, Poland, Romania, Russia (Central European Territory, South European Territory), Slovakia, Spain (mainland), United Kingdom.

## Discussion

Twenty-five species of Sarcophagidae are newly recorded from Croatia as part of this study. Most of these are widely distributed in Europe, which shows that the flesh fly fauna of Croatia is still superficially known. Twenty of them were collected from single localities only. Sarcophaga (Sarcophaga) croatica was the most abundant species in this study and was most common in the Pannonian-Peripannonian region. It was collected in a variety of habitats, from forest edges and grassland to river and lake banks and the seashore in the Mediterranean region. Sarcophaga (Sarcophaga) lehmanni was the second most abundant species and was mainly collected in wet grasslands, forest edges and open habitats (grasslands, agricultural land), showing broad habitat preferences. Similar habitat preferences were observed for S. (S.) lehmanni in Poland (Draber-Mońko 1998; Szpila 1999; Szpila et al. 2015). Sarcophaga (Parasarcophaga) albiceps was the third most numerous species and was mostly collected in grasslands around ponds in the locality of Zmajevac in the Pannonian-Peripannonian region and near freshwater lakes in the Mediterranean region. This confirms a preference for open habitats, as this species was also one of the most abundant species in grasslands in Poland (Szpila et al. 2015). Sixteen of the newly-recorded species, *Amobiapelopei*, *Craticulinatabaniformis*, *Macronychiastriginervis*, *Metopiacampestris*, *Miltogrammaiberica*, *M.punctata*, *Oebalia cylindrica*, *Phyllotelespictipennis*, *Senotainiaconica*, *Taxigrammahilarella*, *T.stictica*, Blaesoxipha (Blaesoxipha) plumicornis, S. (Heteronychia) amita, S. (H.) ancilla, S. (H.) pseudobenaci, and S. (Myorhina) lunigera, were already known from the neighbouring Bosnia and Herzegovina, Hungary or Serbia (Pape 1996, 2004). Besides being a new country record, that of S. (Helicophagella) okaliana is also just the second record from Southeast Europe. Of the 105 species examined during this study, the following have a limited area of distribution: *Sphenometopavariegata*, B. (Blaesoxipha) aurulenta, S. (H.) croca, S. (H.) giganta, S. (S.) adriatica, S. (S.) croatica, S. (S.) pagensis and S. (Thyrsocnema) platariae. Until now, these eight species have been recorded in one to three European countries only, and four of them were described based on specimens collected in Croatia (Baranov 1939, 1941b; Povolný 1987; Pape 1996). The other species recorded in this study are widely distributed throughout Europe and beyond (Pape 1996, 2004). The 57 species recorded by Baranov (1928, 1929, 1930, 1931, 1938, 1939, 1940, 1941a, 1941b, 1942, 1943) and the 18 species recorded by Strukan (1964, 1967, 1968, 1970) were confirmed in this study. Our recent identifications and examination of the literature have enabled us to update the checklist of Croatian flesh flies to 148 species (including two left unnamed). The several new country records indicate that further studies of the flesh fly fauna of Croatia are necessary.
